# Atomically accurate de novo design of antibodies with RFdiffusion

**DOI:** 10.1038/s41586-025-09721-5

**Published:** 2025-11-05

**Authors:** Nathaniel R. Bennett, Joseph L. Watson, Robert J. Ragotte, Andrew J. Borst, DéJenaé L. See, Connor Weidle, Riti Biswas, Yutong Yu, Ellen L. Shrock, Russell Ault, Philip J. Y. Leung, Buwei Huang, Inna Goreshnik, John Tam, Kenneth D. Carr, Benedikt Singer, Cameron Criswell, Basile I. M. Wicky, Dionne Vafeados, Mariana Garcia Sanchez, Ho Min Kim, Susana Vázquez Torres, Sidney Chan, Shirley M. Sun, Timothy T. Spear, Yi Sun, Keelan O’Reilly, John M. Maris, Nikolaos G. Sgourakis, Roman A. Melnyk, Chang C. Liu, David Baker

**Affiliations:** 1 Department of Biochemistry, University of Washington, Seattle, WA USA; 2 Institute for Protein Design, University of Washington, Seattle, WA USA; 3 Graduate Program in Molecular Engineering, University of Washington, Seattle, WA USA; 4 Department of Bioengineering, University of Washington, Seattle, WA USA; 5 Department of Biomedical Engineering, University of California, Irvine, CA USA; 6 Center for Synthetic Biology, University of California, Irvine, CA USA; 7 Department of Pharmaceutical Sciences, University of California, Irvine, CA USA; 8 Department of Pediatrics, Children’s Hospital of Philadelphia, Philadelphia, PA USA; 9 Department of Pediatrics, Perelman School of Medicine at the University of Pennsylvania, Philadelphia, PA USA; 10 Howard Hughes Medical Institute, University of Washington, Seattle, WA USA; 11 Molecular Medicine Program, The Hospital for Sick Children, Toronto, Ontario Canada; 12 Center for Biomolecular and Cellular Structure, Institute for Basic Science (IBS), Daejeon, Republic of Korea; 13 Department of Biological Sciences, Korea Advanced Institute of Science and Technology (KAIST), Daejeon, Republic of Korea; 14 Graduate Program in Biological Physics, Structure and Design, University of Washington, Seattle, WA USA; 15 Center for Computational and Genomic Medicine, Department of Pathology and Laboratory Medicine, Children’s Hospital of Philadelphia, Philadelphia, PA USA; 16 Department of Biochemistry and Biophysics, University of Pennsylvania, Philadelphia, PA USA; 17 Division of Oncology and Center for Childhood Cancer Research, Children’s Hospital of Philadelphia, Philadelphia, PA USA; 18 Department of Biochemistry, University of Toronto, Toronto, Ontario Canada; 19 Department of Chemistry, University of California, Irvine, CA USA; 20 Department of Molecular Biology & Biochemistry, University of California, Irvine, CA USA

**Keywords:** Protein design, Cryoelectron microscopy

## Abstract

Despite the central role of antibodies in modern medicine, no method currently exists to design novel, epitope-specific antibodies entirely in silico. Instead, antibody discovery currently relies on immunization, random library screening or the isolation of antibodies directly from patients^1^. Here we demonstrate that combining computational protein design using a fine-tuned RFdiffusion^2^ network with yeast display screening enables the de novo generation of antibody variable heavy chains (VHHs), single-chain variable fragments (scFvs) and full antibodies that bind to user-specified epitopes with atomic-level precision. We experimentally characterize VHH binders to four disease-relevant epitopes. Cryo-electron microscopy confirms the binding pose of designed VHHs targeting influenza haemagglutinin and *Clostridium difficile* toxin B (TcdB). A high-resolution structure of the influenza-targeting VHH confirms atomic accuracy of the designed complementarity-determining regions (CDRs). Although initial computational designs exhibit modest affinity (tens to hundreds of nanomolar *K*_d_), affinity maturation using OrthoRep^3^ enables production of single-digit nanomolar binders that maintain the intended epitope selectivity. We further demonstrate the de novo design of scFvs to TcdB and a PHOX2B peptide–MHC complex by combining designed heavy-chain and light-chain CDRs. Cryo-electron microscopy confirms the binding pose for two distinct TcdB scFvs, with high-resolution data for one design verifying the atomically accurate design of the conformations of all six CDR loops. Our approach establishes a framework for the computational design, screening and characterization of fully de novo antibodies with atomic-level precision in both structure and epitope targeting.

## Abstract (web summary)

The combination of computational design, laboratory-based screening and biophysical validation enables the de novo generation of variable heavy-chain antibody fragments and antibodies that precisely target chosen disease-related molecules.

## Main

Antibodies are the dominant class of protein therapeutics, with over 160 antibody therapeutics currently licensed globally and a market value expected to reach US$445 billion in the next 5 years^4^. Antibody development generally proceeds in two stages: (1) the discovery of antibodies that bind to a specific epitope; and (2) the subsequent affinity maturation and clinical optimization of those antibodies. Currently, identifying epitope-specific antibodies relies on animal immunization or screening of antibody libraries to identify candidate molecules that bind to a desired target, followed by subsequent epitope mapping. These methods are laborious, time-consuming and can fail to identify antibodies that interact with the therapeutically relevant epitope^1^. Efforts at computational design of antibodies have generally focused on the second optimization step of antibody development, such as sampling alternative native CDR loops to improve affinities^5,6^ or using Rosetta^7^ sequence design to improve the interacting regions. More recently, structure-based and sequence-based deep learning networks have been trained to design novel antibody sequence variants^8–10^, but these methods require an initial binding antibody from which to optimize. There have also been recent advances in antibody optimization with deep learning methods trained on data generated by powerful new experimental methods^11,12^. By contrast, computational methods able to perform the first stage of antibody design (generating epitope-specific binding antibodies) do not exist, and de novo (no homology to an existing antibody targeting that epitope) design of antibodies therefore remains an unsolved problem. There has been rapid progress in designing binding proteins (not antibodies) using RFdiffusion^2,13,14^. However, as with other methods for de novo interface design^15–17^, these binders almost exclusively rely on regular secondary structure-based (helical or strand) interactions with the target epitope, and the original (‘vanilla’) RFdiffusion network is therefore unable to design antibodies de novo (Supplementary Fig. 1; see ref. ^18^).

An ideal method for designing de novo antibodies would enable: (1) targeting of any specified epitope on any target of interest; (2) focusing of sampling on the CDR loops, keeping the framework sequence and structure close to a user-specified highly optimized therapeutic antibody framework; and (3) sampling of alternative rigid-body placements of the designed antibody with respect to the epitope. We hypothesized that a specialized version of RFdiffusion fine-tuned on antibody structures should be capable of designing de novo CDR-mediated interfaces, given the diversity and quality of de novo interfaces that RFdiffusion can design and given that the underlying thermodynamics of interface formation are the same, and set out to develop such a method.

## Training RFdiffusion for antibody design

RFdiffusion uses the AlphaFold2 (ref. ^19^) and RF2 frame representation of protein backbones comprising the Cα coordinate and N-Cα-C rigid orientation for each residue. During training, a noising schedule is used that, over a set number of ‘timesteps’ (*T*), corrupts the protein frames towards random prior distributions (Cα coordinates are corrupted with three-dimensional Gaussian noise, and residue orientations with Brownian motion on SO3). During training, a Protein Data Bank (PDB) structure and a random timestep (*t*) are sampled, and *t* noising steps are applied to the structure. RFdiffusion predicts the de-noised (*pX*_0_) structure at each timestep, and a mean squared error loss is minimized between the true structure (*X*_0_) and the prediction (*pX*_0_). At inference time, a random residue distribution (*X*_*T*_) is sampled, and RFdiffusion iteratively de-noises this to generate novel protein structures.

**Fig. 1:**
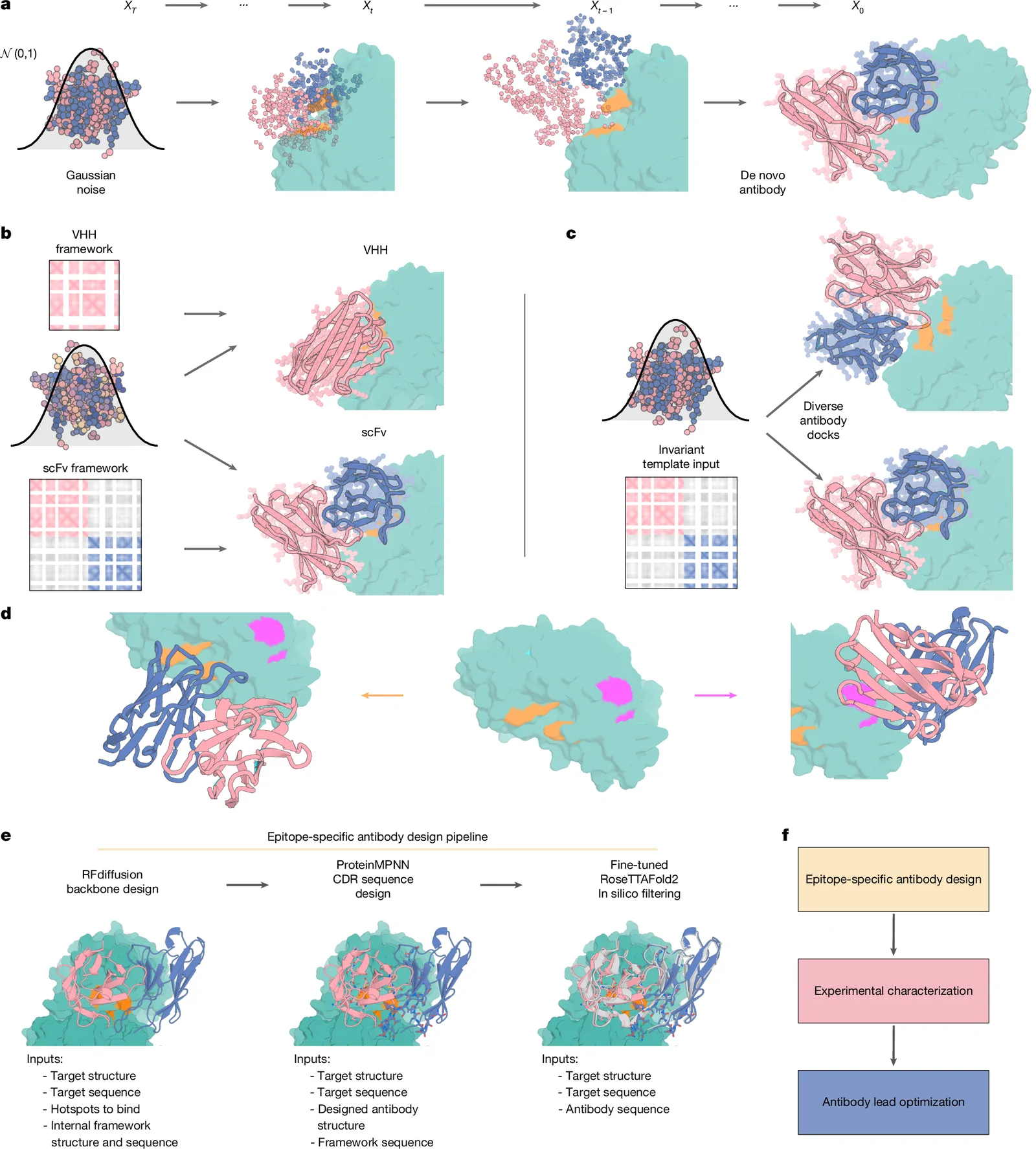
Overview of RFdiffusion for antibody design. **a**, RFdiffusion is trained such that at time *T*, a sample is drawn from the prior distribution (three-dimensional Gaussian distribution for translations and uniform SO3 distribution for rotations), and de-noised between times *T* and 0 to generate an (in this case) scFv. **b**, The antibody framework is provided as a sequence and ‘template’ to RFdiffusion; the latter specifying the pairwise distances and dihedral angles between framework residues. For example, one can specify the design of a VHH (top) or scFv (bottom). **c**, Diversity in the antibody–target dock is achieved because the framework template does not encode the rigid body framework–target relationship. Diverse docking modes are sampled by RFdiffusion. **d**, The epitope is specified by provision of ‘hotspot’ residues, which direct the designed antibody (compare orange, left, versus pink, right). **e**, Overview of the computational design pipeline described in this article. RFdiffusion performs the backbone design step, given a target, epitope hotspots and antibody framework. ProteinMPNN designs only the sequence of the CDR residues (not the framework residues). Fine-tuned RoseTTAFold2 predicts the structure of the designed antibody, given the target (sequence, structure and, optionally, some fraction of hotspot residues) and designed antibody sequence. Self-consistency (high similarity between predicted and designed structures) and high confidence (low predicted alignment error) define in silico success. Note that AlphaFold3, not available at the time of this work, is a better predictor of success than RoseTTAFold2. **f**, The contribution of this work is the epitope-specific antibody design pipeline depicted in panel **e**. Several methods can be used to experimentally validate designs and subsequently affinity-mature or optimize them. In this work, we used yeast surface display and/or *E. coli* expression with SPR for experimental validation (taking approximately 6 weeks and 2 weeks post-oligonucleotide order, respectively), and OrthoRep affinity maturation.

We fine-tuned RFdiffusion predominantly on antibody complex structures (Fig. 1; see Methods in Supplementary Information). At each step of training, the antibody structure is corrupted. To permit specification of the framework structure and sequence at inference time, the framework sequence and structure are provided as conditioning input to RFdiffusion during training (Fig. 1b). Because it is desirable for the rigid-body position (dock) between antibody and target to be designed by RFdiffusion along with the CDR loop conformations, the framework structure is provided in a global-frame-invariant manner during training (Fig. 1c). We utilize the ‘template track’ of RF2/RFdiffusion to provide the framework structure as a two-dimensional matrix of pairwise distances and dihedral angles between each pair of residues (a representation from which three-dimensional structures can be accurately recapitulated)^20^ (Supplementary Fig. 1a). The framework and target templates do not encode their relative positions in the three-dimensional space. In this work, we kept the sequence and structure of the framework region fixed, and focused on the design of the CDRs and the overall rigid-body placement of the antibody to the target. We trained RFdiffusion with an additional one-hot encoded ‘hotspot’ feature, which provides some fraction of the residues that the antibody CDRs interact with, such that at inference, we can direct antibodies towards a specific site (Fig. 1d; we refer to these sites as ‘epitopes’ throughout the remainder of the text). For simplicity, we refer to this fine-tuned version of the network as RFdiffusion for the remainder of this paper.

**Extended Data Fig. 1:**
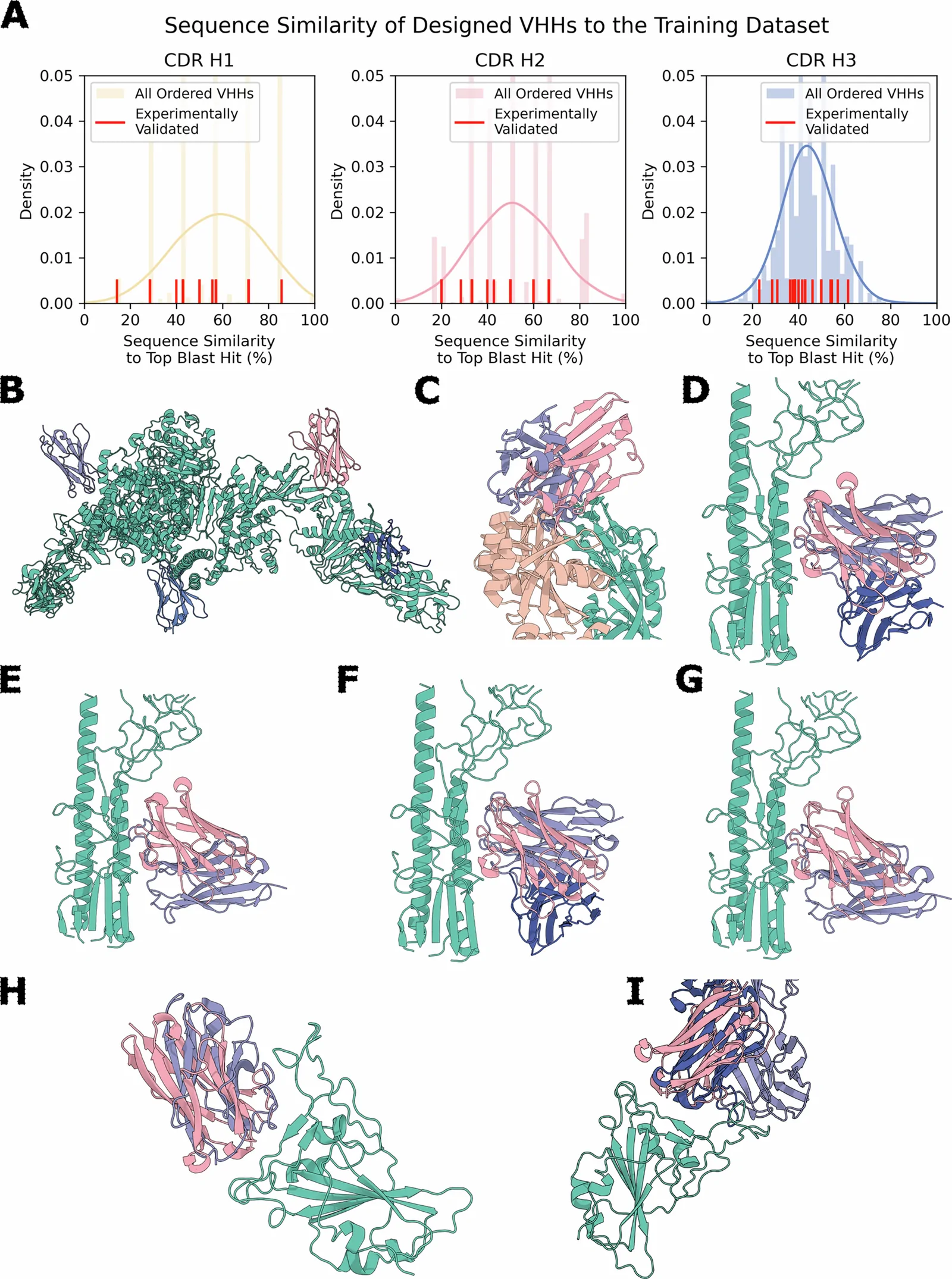
Designed VHH are dissimilar to the training dataset. **A**) Designed VHH sequences are distinct from the training dataset. Blastp^49^ was used to find hits against the SAbDab^50^, and the similarity of the CDR loops in the top blast hit were reported for all VHHs experimentally tested in this study. Note also that the 28 VHHs confirmed to bind their targets by SPR do not show enhanced similarity to the training set (red lines). **B-I**) Designed VHH are structurally distinct from immunoglobulins binding (when existent) to the epitopes used in this study. For each of the highest affinity VHHs identified for each target, and the structurally characterized influenza HA VHH, the closest complex in the PDB is shown. Designed VHHs (pink) are shown in complex with their designed target (teal and tan). The closest complex was identified visually (Methods). **B**) Designed TcdB VHH aligned against 3 VHHs from PDB ID: 6OQ5 (shades of blue). The designed TcdB VHH binds to a site for which no antibody or VHH structure exists in the PDB. **C**) Designed RSV Site III VHH aligned against VHH from PDB ID: 5TOJ (blue). **D**) Highest affinity designed influenza HA VHH aligned against Fv from PDB ID: 8DIU (shades of blue). **E**) Highest affinity designed influenza HA VHH aligned against VHH from PDB ID: 6FYT (blue). **F**) Structurally characterized (cryoEM) designed influenza HA VHH aligned against Fv from PDB ID: 8DIU (shades of blue). **G**) Structurally characterized (cryoEM) designed influenza HA VHH aligned against VHH from PDB ID: 6FYT (blue). **H**) Designed SARS-CoV-2 VHH aligned against VHH from PDB ID: 8Q94 (blue). **I**) Designed SARS-CoV-2 VHH aligned against FAb from PDB ID: 7FCP (shades of blue).

With this training regime, RFdiffusion is able to design antibody structures that closely match the structure of the input framework structure and target the specified epitope with novel CDR loops (Supplementary Fig. 1). After the RFdiffusion step, we use ProteinMPNN to design the CDR loop sequences. The designed antibodies make diverse interactions with the target epitope and differ significantly from sequences in the training dataset (Extended Data Fig. 1). There was no correlation between training dataset similarity and binding success (Extended Data Fig. 1a, red lines).

## Fine-tuning RF2 for antibody validation

Design pipelines typically produce a wide range of solutions to any given design challenge. An effective way to filter designed proteins and interfaces that are most likely to succeed experimentally is based on the similarity of the designed structure to the AlphaFold2-predicted structure for the designed sequence (this is often referred to as ‘self-consistency’), which has been shown to correlate well with experimental success^21,22^. In the case of antibodies, however, AlphaFold2 fails to accurately predict antibody–antigen structures^23^, preventing its use as a filter in an antibody design pipeline, and at the outset of this project, AlphaFold3 (ref. ^24^) was not available.

**Extended Data Fig. 2:**
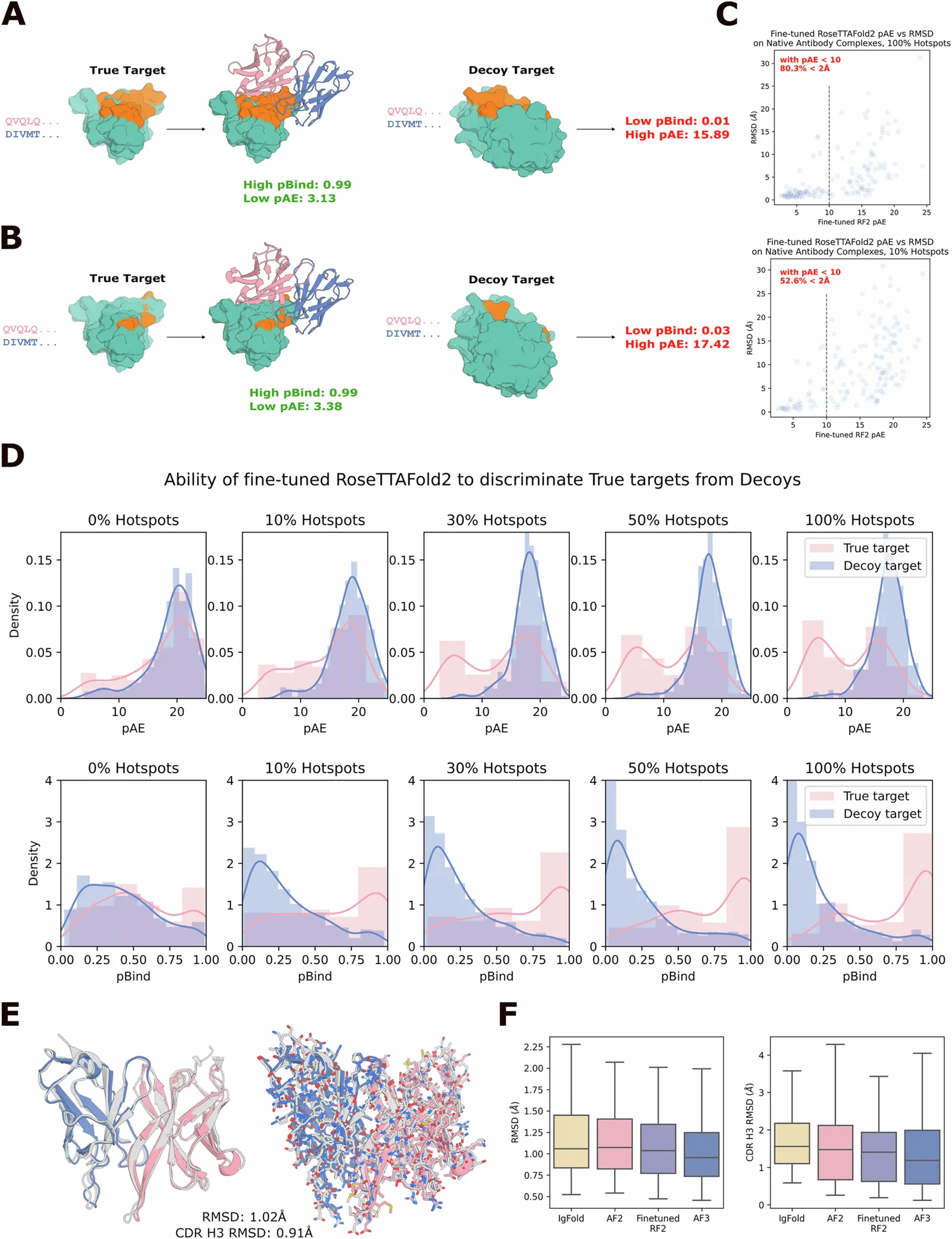
Fine-tuned RoseTTAFold2 can distinguish true complexes from decoy complexes. **A**-**B**) Cherry-picked example of RoseTTAFold2 correctly distinguishing a “true” from a “decoy” complex. The sequence of antibody 7Y1B was provided either with the correct (PDB ID: 7Y1B) or decoy (PDB ID: 8CAF) target. Both with 100% (**A**) or 10% (**B**) of “hotspots” provided, RF2 near-perfectly predicts binding (top row) or non-binding (bottom row). **C**) Fine-tuned RoseTTAFold2 reliably predicts its own accuracy. Correlation between RF2 predicted aligned error (pAE) and RMSD to the native structure with 100% (top) or 10% (bottom) of “hotspot” residues provided. With mean pAE <10, 80.3% of structures are within 2 Å when 100% of “hotspots” are provided (along with the holo target structure), with this falling to 52.6% when only 10% of hotspots are provided. **D**) Quantification of the fine-tuned RF2’s ability to distinguish true targets from decoy targets with both pAE (top row) and pBind (bottom row). Note that this ability depends on the proportion of “hotspots” provided. Without any “hotspots” provided, RF2 is hardly predictive, because RF2 without privileged information is quite rarely confident or accurate in its antibody complex predictions. **E-F**) Fine-tuned RoseTTAFold is also performant at antibody monomer prediction. 86 antibodies released after the RF2, AlphaFold and IgFold training dataset date cutoff (January 13th, 2023) that share <30% target sequence similarity to any antibody complex released prior to this date were predicted as monomers with either fine-tuned RF2, IgFold, AF2 and AF3. **E**) The median Fv quality prediction (by overall RMSD) of fine-tuned RF2, of (PDB ID: 8GPG), with (right) and without (left) sidechains shown (gray: native; colors: prediction). While the backbone RMSD is close to the true structure, some sidechains are incorrectly positioned. **F**) Fine-tuned RF2 slightly outperforms IgFold at prediction accuracy. Overall prediction accuracy is slightly improved in fine-tuned RF2 vs IgFold (*p* = 0.026, Student’s Paired T-test), with greater improvements in CDR H3 prediction accuracy (*p* = 0.0003, Student’s Paired T-test).

We sought to improve design filtering by fine-tuning RoseTTAFold2 on antibody structures. To simplify antibody structure prediction, we provided information during training about the structure of the target and the location of the target epitope to which the antibody binds; the fine-tuned RF2 must still correctly model the CDRs and find the correct orientation of the antibody to the targeted region. The rationale for providing this information is that the target structure and binding location are available during design (but are typically not available during general structure prediction). With this training regimen and additional information, RF2 is able to robustly distinguish true antibody–antigen pairs from decoy pairs and often accurately predicts antibody–antigen complex structures, but only when the bound (holo) conformation of the target structure and epitope information is provided (Extended Data Fig. 2a–d). At monomer prediction, the fine-tuned RF2 outperformed previous models available at the time, especially at CDR H3 structure prediction (Extended Data Fig. 2e,f).

**Extended Data Fig. 3:**
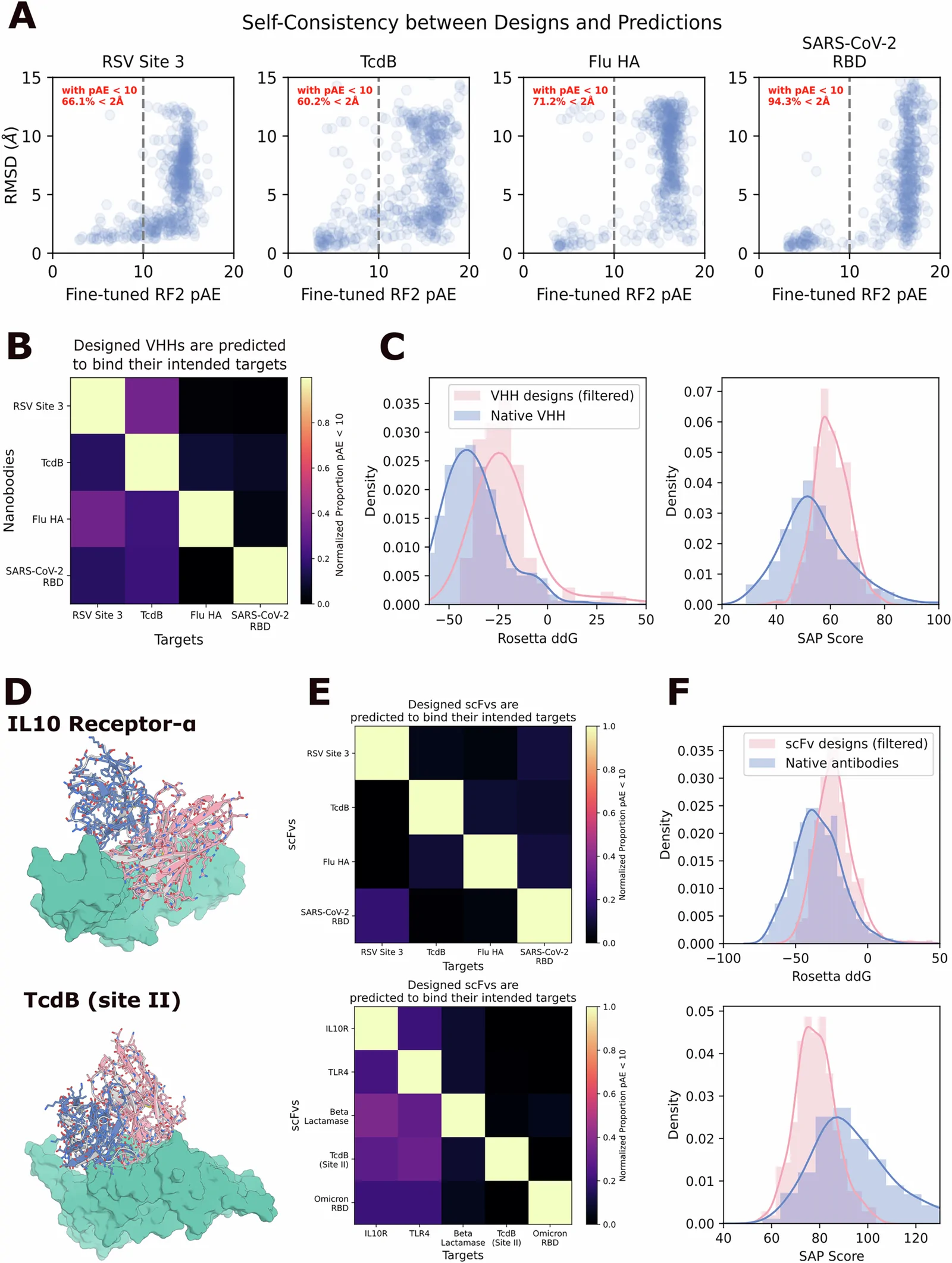
Fine-tuned RoseTTAFold2 recapitulates design structures and computationally demonstrates specificity of VHHs for their targets. **A-C: VHH Design. A**) Comparison of RF2 pAE and RMSD of the prediction to the design model. A significant fraction of designs are re-predicted by RF2 (given 100% of “hotspots”), and pAE correlates well with accuracy to the design model. **B**) RF2 can be used to assess quality of designed VHHs. Providing the VHH sequence with the true target structure (used during design) leads to higher rates of high-confidence predictions than predicting the same sequence with a decoy structure (not used in design), as assessed by the fraction of predictions with pAE <10 (normalized to the fraction of predictions with pAE <10 for that target with its “correct” VHH partners). In these experiments, the true or decoy target was provided along with 100% of hotspot residues, with those hotspot residues derived from the target with its “true” designed VHH bound. **C**) Orthogonal assessment of designed VHHs with Rosetta demonstrates that the interfaces of RF2-approved (RMSD < 2 Å to design model, pAE <10) VHH designs have estimated binding energy (ddG) only slightly less favourable than native VHH (left) and slightly higher spatial aggregation propensity (SAP) score as compared to natives (right). **D-F: scFv design**. **D**) RFdiffusion was used to generate scFv designs using the framework from Herceptin (hu4D5-8), which has been used to make scFvs previously^51^. Five targets were chosen (IL10 Receptor-α, TLR4, β-lactamase, TcdB and SARS-CoV-2 (omicron) RBD (PDB IDs: 6X93, 4G8A, 4ZAM, 7ML7, 7WPC). Shown are two examples with close agreement between the design model and the fine-tuned RF2 prediction (RMSD (Å): 0.60, 0.43; pAE: 4.73, 3.52). Gray: designs, Pink: RF2 prediction. **E**) Top: Against the same four targets to which VHHs were successfully designed, fine-tuned RF2 also predicts good specificity of designed scFvs to the designed target vs decoy targets. Bottom: against the set of five aforementioned targets, fine-tuned RF2 similarly predicts high scFv specificity to the designed target vs decoy targets. **F**) Orthogonal assessment of designed scFvs with Rosetta demonstrates that the interfaces of RF2-approved (RMSD < 2 Å to design model, pAE <10) scFv designs have low ddG (top; only slightly worse than native Fabs) and lower spatial aggregation propensity (SAP) score as compared to natives (bottom).

When this fine-tuned RF2 network is used to re-predict the structure of RFdiffusion-designed VHHs, a significant fraction are confidently predicted to bind in an almost identical manner to their designed structure (Extended Data Fig. 3a). Furthermore, in silico cross-reactivity analyses demonstrated that RFdiffusion-designed VHHs are rarely predicted to bind to unrelated proteins (Extended Data Fig. 3b). VHHs that are confidently predicted to bind to their designed target are predicted to form high-quality interfaces, as measured by Rosetta ddG (Extended Data Fig. 3c). This indicates that RF2 filtering might enrich for experimentally successful binders.

## Design and characterization of VHHs

We initially focused on the design of single-domain antibodies (VHHs) produced by camelids^25^. To date, two VHH-based therapies have been approved by the FDA with many clinical trials ongoing^25^. Despite having fewer CDR loops (three) than conventional antibodies (six), the average interaction surface area of a VHH is very similar to that of an antibody^26^, suggesting that a method capable of VHH design could also be suitable for antibody design. Indeed, in silico metrics for scFvs and VHHs showed similar qualities of interfaces, as assessed by Rosetta^7^ and fine-tuned RF2 (Extended Data Fig. 3b–f).

We chose a widely used humanized VHH framework (h-NbBcII10FGLA)^27^ as the basis of our VHH design campaigns, and designed VHHs to a range of disease-relevant targets: *C. difficile* TcdB, influenza H1 haemagglutinin, respiratory syncytial virus (RSV) sites I and III, SARS-CoV-2 receptor-binding domain (RBD) and IL-7Rα. Computationally filtered designs were screened either at high throughput by yeast surface display (9,000 designs per target; RSV sites I and III, RBD and influenza haemagglutinin) or at lower throughput with *Escherichia coli* expression and single-concentration surface plasmon resonance (SPR; 95 designs per target; TcdB, IL-7Rα and influenza haemagglutinin; the latter was screened using both methods).

**Fig. 2:**
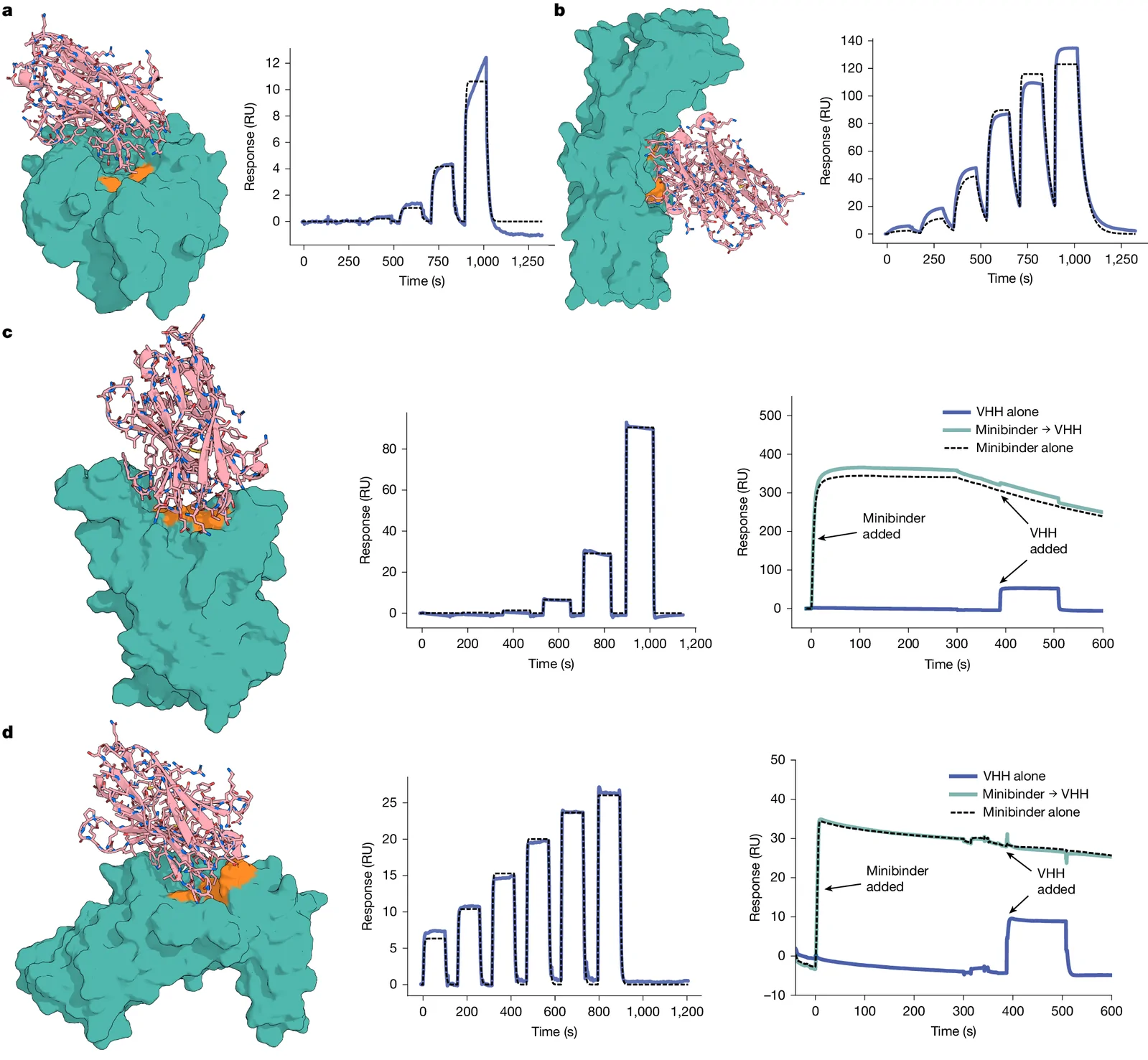
Biochemical characterization of designed VHHs. **a**,**b**, Nine thousand designed VHHs were screened against RSV site III (**a**; VHH_RSV_01) and influenza haemagglutinin (**b**; VHH_flu_01) with yeast surface display, before soluble expression of the top hits in *E. coli*. SPR demonstrated that the highest affinity VHHs to RSV site III and influenza haemagglutinin bound their respective targets with 1.4 μM and 78 nM, respectively. **c**, Nine thousand VHH designs were tested against the SARS-CoV-2 RBD, and after soluble expression, SPR confirmed an affinity of 5.5 μM to the target for design VHH_RBD_D4 (left). Binding was to the expected epitope, confirmed by competition with a structurally confirmed de novo binder (AHB2 (PDB ID 7UHB), right). **d**, Ninety-five VHH designs were tested against *C. difficile* TcdB. The highest affinity VHH, VHH_TcdB_H2, bound with 262 nM affinity (left), and also competed with a structurally confirmed de novo binder (FZD48, PDB ID 9CM5 (ref. ^29^)) to the same epitope (right). See also Extended Data Fig. 4a–c for quantification of the competition shown in panels **c**,**d**. For all panels, the measured binding response is indicated in a solid blue line, and the global fit using a 1:1 binding interaction model is indicated with a black dashed line.

**Extended Data Fig. 4:**
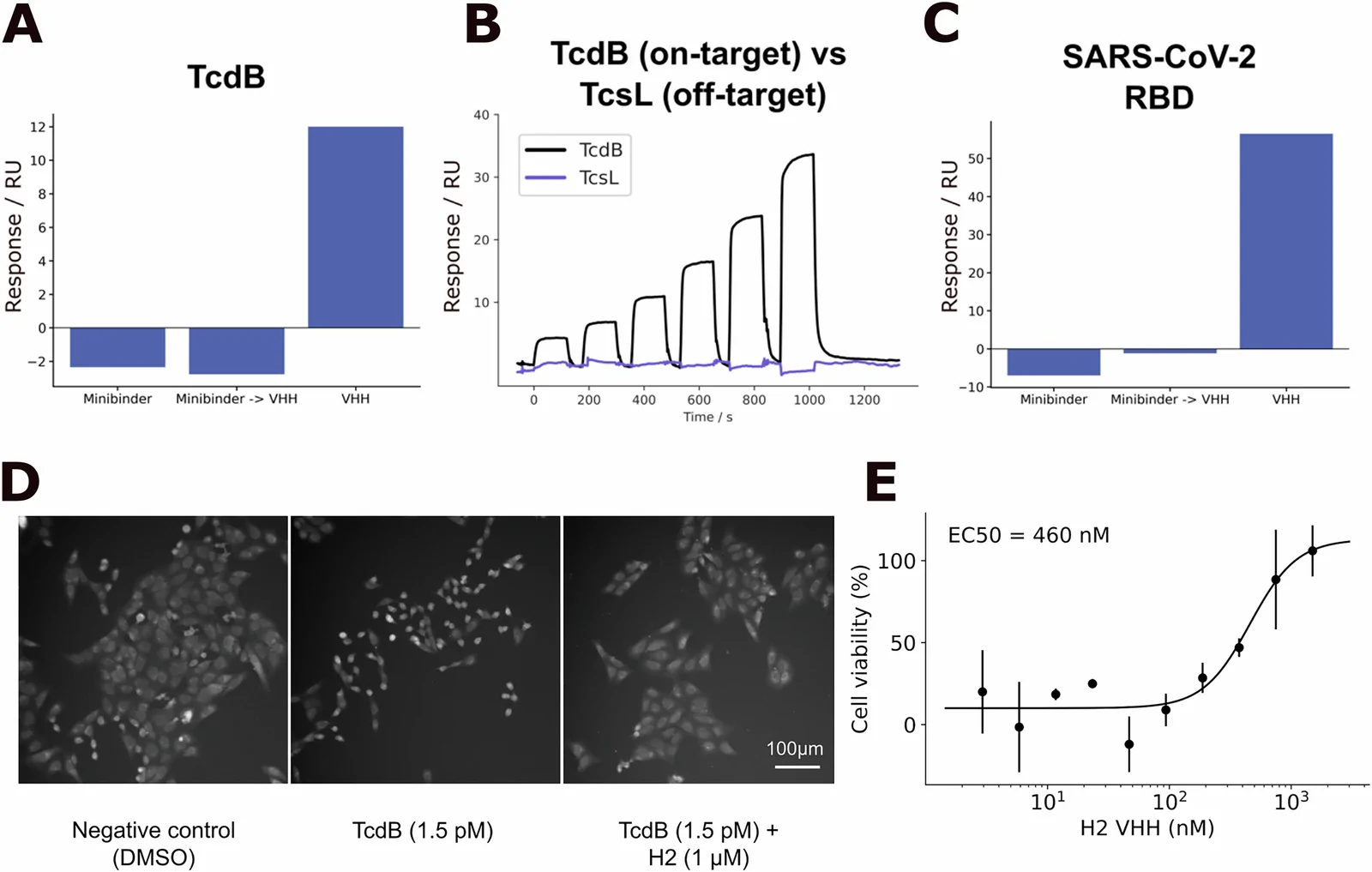
Analysis of SPR Competition Assays. **A**) TcdB VHH binds to the correct epitope. The average response during VHH injection normalized to the response immediately preceding VHH injection for TcdB VHH competition with minibinder fzd48. When the minibinder is injected prior to the VHH (middle bar), the VHH no longer binds, confirming competition with the minibinder. **B**) TcdB VHH is specific. No binding is observed to the closely related *Clostridium sordellii* TcsL toxin, indicating that it is binding through specific interactions. **C**) SARS-CoV-2 RBD VHH competition with the minibinder AHB2. As in (**A**), when minibinder is injected prior to the VHH injection (middle bar) no response is observed. When the VHH is injected without a preceding minibinder injection (right bar), the VHH binds as expected. (**A**) and (**C**) are the quantification from the rightmost panels of Fig. 2a,b. **D**) Brightfield microscopy of Vero CSPG4 KO cells treated with vehicle, TcdB alone or TcdB + VHH after 24 h. **E**) Neutralization of TcdB by VHH_TcdB_H2 in CSPG4 KO Vero cells. Cell viability is measured after 48 h. Points indicate the mean and error bars are the standard deviation across two independent replicates.

The highest affinity binders to RSV site III, influenza haemagglutinin, RBD and TcdB are shown in Fig. 2a–c,e, respectively (see also Supplementary Fig. 2 for all the SPR traces of confirmed VHH binders identified in this study and Supplementary Methods Table 6 for success rates against each target, which range from 0% to 2%). The CDR loops are distinct from VHHs observed in nature, indicating substantial generalization beyond the training dataset (Extended Data Fig. 1). Of the haemagglutinin binders tested against the insect-cell-produced haemagglutinin monomer, the highest affinity binder had a dissociation constant (*K*_d_) of 78 nM (Fig. 2b), with other binders having affinities of 546 nM, 698 nM and 790 nM. For TcdB, the target epitope was the Frizzled interface, for which there are no antibodies or VHHs targeting this site in the PDB. For the best-designed VHH from both RBD (*K*_d_ = 5.5 μM; Fig. 2c) and TcdB (*K*_d_ = 260 nM; Fig. 2d), binding was confirmed to be to the desired epitope: binding was completely abolished upon addition of a previously designed, structurally characterized de novo binder to that epitope (AHB2 (PDB ID 7UHB^28^) for RBD and FZD48 (PDB ID 9CM5 (ref. ^29^)) for TcdB; Fig. 2c,d and Extended Data Fig. 4a–c). This TcdB VHH also neutralized TcdB toxicity in *CSPG4*-knockout cells (an alternative TcdB receptor) with a half-maximal effective concentration (EC_50_) of 460 nM (Extended Data Fig. 4d,e). For TcdB, the interactions were specific, with no binding observed to the highly related (70% sequence homology) *Paeniclostridium sordellii* lethal toxin L (TcsL; Extended Data Fig. 4b). These data demonstrate the ability of RFdiffusion to design VHHs that make specific interactions with the target epitope.

## Cryo-EM of a VHH-binding influenza haemagglutinin

**Fig. 3:**
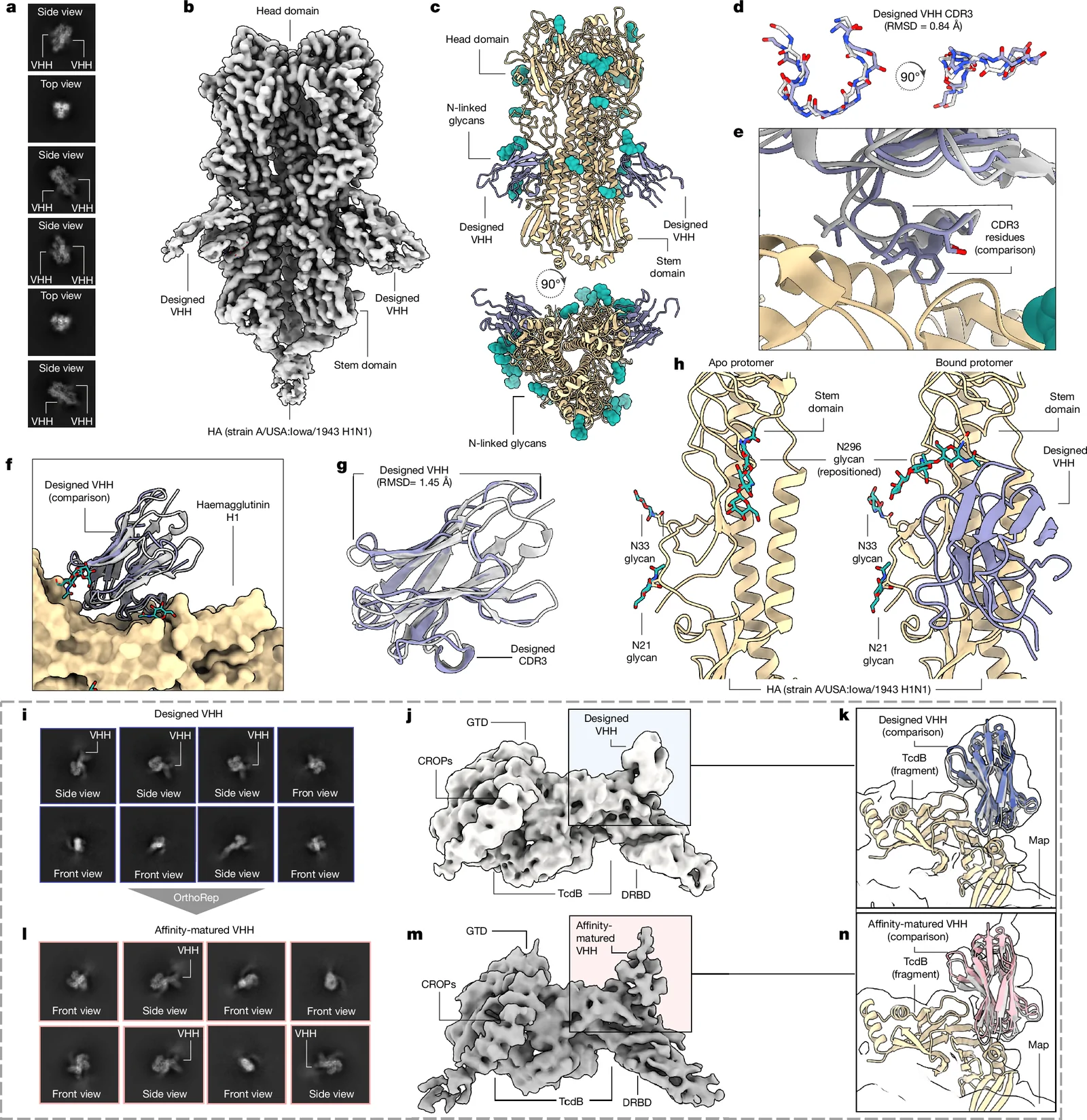
Cryo-EM structural characterization of de novo-designed VHH binding to influenza haemagglutinin and TcdB. **a**, Labelled cryo-EM two-dimensional class averages of designed VHH_flu_01 bound to influenza haemagglutinin (HA) strain A/USA:Iowa/1943 H1N1. **b**, The 3.0 Å cryo-EM three-dimensional reconstruction shows VHH_flu_01 bound to H1 along the stem in two protomers. **c**, Cryo-EM structure of VHH_flu_01 bound to influenza haemagglutinin. **d**, Superposition of the designed VHH CDR3 structure with the cryo-EM structure. **e**, Comparison of predicted CDR3 rotamers compared with the built 3.0 Å cryo-EM structure. **f**,**g**, The cryo-EM structure closely matches the design. **h**, Examination of apo haemagglutinin protomers juxtaposed with those bound to the designed VHH shows repositioning of glycan N296 to allow for binding of the designed VHH to the stem. **i**, Labelled cryo-EM two-dimensional class averages of the designed VHH, VHH_TcdB_H2, bound to full-length TcdB. **j**, The 4.6 Å cryo-EM three-dimensional reconstruction of the complex shows VHH_TcdB_H2 bound to the target epitope as predicted. CROPs, combined repetitive oligopeptides; GTD, glucosyltransferase domain. **k**, Owing to the modest resolution, a fragment of TcdB was first docked into the cryo-EM density map, and the full design model—including both the TcdB fragment and the designed VHH—was then aligned to the pre-fitted TcdB fragment. The predicted design closely matches the experimentally determined complex in structure, epitope targeting and overall conformation. **l**, Labelled cryo-EM two-dimensional class averages of the designed VHH, VHH_TcdB_H2_ortho, bound to full-length TcdB. **m**, The 5.7 Å cryo-EM three-dimensional reconstruction of the complex shows that VHH_TcdB_H2_ortho bound the target epitope as predicted. **n**, A TcdB fragment was docked into the cryo-EM map, followed by alignment of the full model including the OrthoRep-matured VHH. The resulting structure shows no detectable change in binding orientation or docking angle compared with the original design, indicating that OrthoRep maturation preserved the predicted mode of epitope engagement. In all panels: yellow indicates haemagglutinin; grey denotes the computational design prediction; pink or navy shows VHH (cryo-EM); and teal indicates glycan.

**Extended Data Fig. 5:**
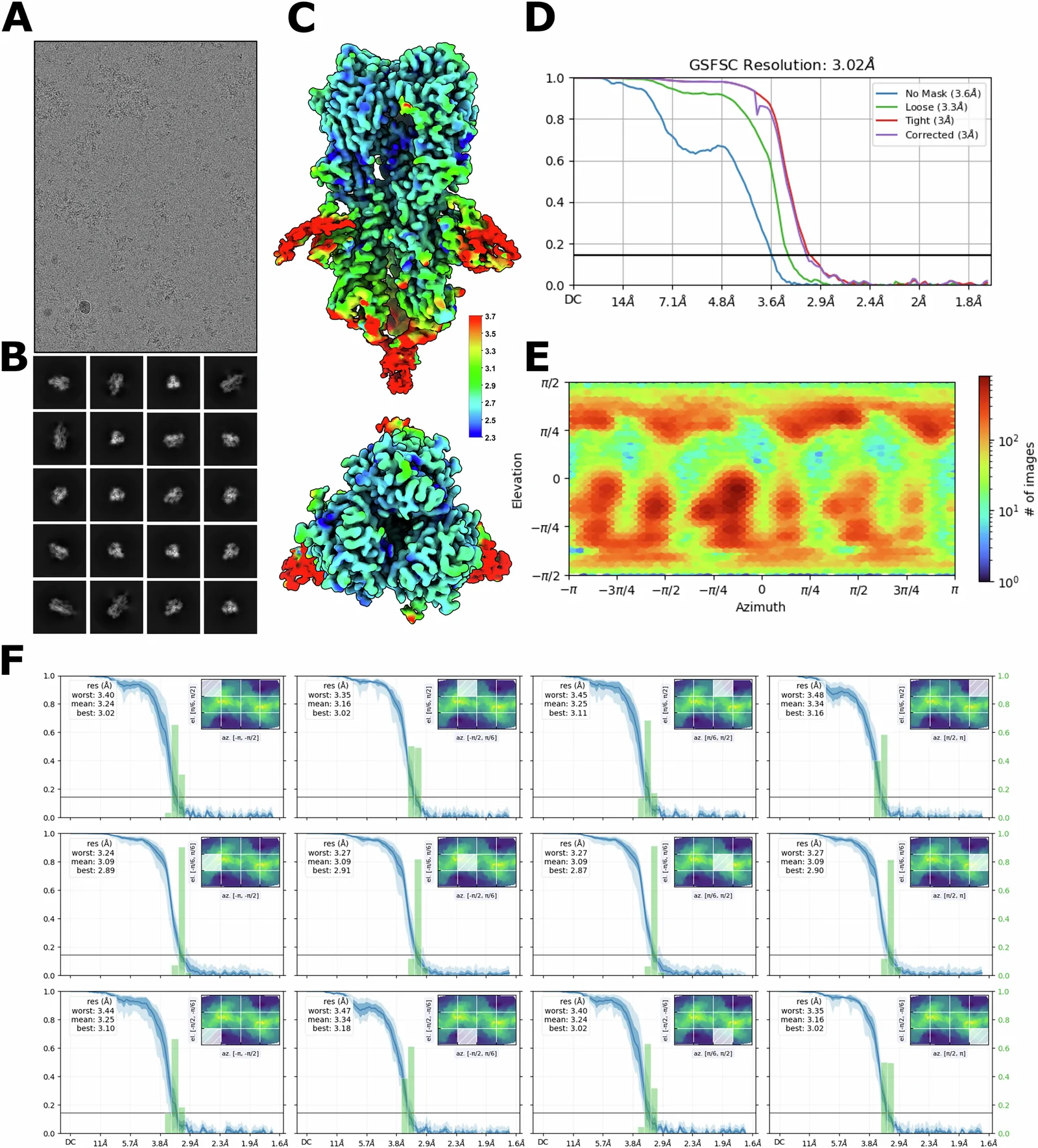
Cryo-EM structure determination statistics for a de novo designed VHH bound to an influenza HA trimer. **A**) Representative raw micrograph showing ideal particle distribution and contrast. **B**) 2D Class averages of Influenza H1+designed VHH with clearly defined secondary structure elements and a full-sampling of particle view angles. **C**) Cryo-EM local resolution map calculated using an FSC value of 0.143 viewed along two different angles. Local resolution estimates range from ~2.3 Å at the core of H1 to ~3.7 Å along the periphery of the designed VHH. **D**) Global resolution estimation plot. **E**) Orientational distribution plot demonstrating complete angular sampling. **F**) Orientational diagnostics data.

We sought to evaluate design accuracy by cryo-electron microscopy (cryo-EM) structure determination of the designed anti-haemagglutinin VHHs in complex with natively glycosylated, trimeric influenza haemagglutinin glycoprotein (strain A/USA:Iowa/1943 H1N1; Supplementary Fig. 4), which retains the conserved stem epitope used during computational VHH design and upstream biochemical screening. Cryo-EM data processing revealed that one VHH design effectively bound to the fully glycosylated haemagglutinin trimer (out of the four tested), denoted hereafter as VHH_flu_01 (Fig. 3 and Extended Data Fig. 5). Two-dimensional classification of all particles in the dataset (Fig. 3a) and the determined 3.0 Å structure of the complex (Fig. 3b and Supplementary Methods Table 10) identified approximately 66% of haemagglutinin particles bound to a maximum of two VHHs per trimer (Fig. 3a–h). This partial occupancy is probably attributable to the N296 glycan, which, in unbound subunits, partially occludes the target epitope but reorients when bound to VHH_flu_01 (see Fig. 3h).

The structure of influenza haemagglutinin bound to two copies of VHH_flu_01 (Fig. 3b,c and Extended Data Fig. 5) reveals a VHH approach angle that closely matches the predicted model (Fig. 3f) and a VHH backbone that is very close to the RFdiffusion design, with a calculated root mean square deviation (RMSD) of 1.45 Å (Fig. 3g). The CDR3 structure is also very similar between the cryo-EM structure and the computational model (RMSD = 0.8 Å; Fig. 3d), with residues V100, V101, S103 and F108 in the de novo-designed CDR3 loop interacting with the influenza haemagglutinin stem epitope in the cryo-EM structure, as designed by RFdiffusion and re-predicted with RF2 (Fig. 3e). The design is highly dissimilar from the closest antibody–VHH binding to this epitope in the PDB (Extended Data Fig. 1f,g and Supplementary Fig. 5). Together, these results demonstrate the VHH design with atomic-level precision.

## Cryo-EM of VHHs to TcdB and SARS-CoV-2

To improve the binding affinity of de novo-designed VHHs, we utilized the orthogonal error-prone DNA replication system, OrthoRep, for continuous hypermutation of target genes in vivo^30,31^. OrthoRep has been shown to drive the rapid affinity maturation of yeast surface-displayed antibodies. We used this capability to affinity-mature VHHs targeting TcdB, influenza H1 haemagglutinin and the SARS-CoV-2 RBD. Affinity-matured VHHs acquired several mutations relative to the parent designs and improved binding affinities by approximately two orders of magnitude (Supplementary Fig. 3), making them suitable candidates for downstream cryo-EM structural characterization.

**Extended Data Fig. 6:**
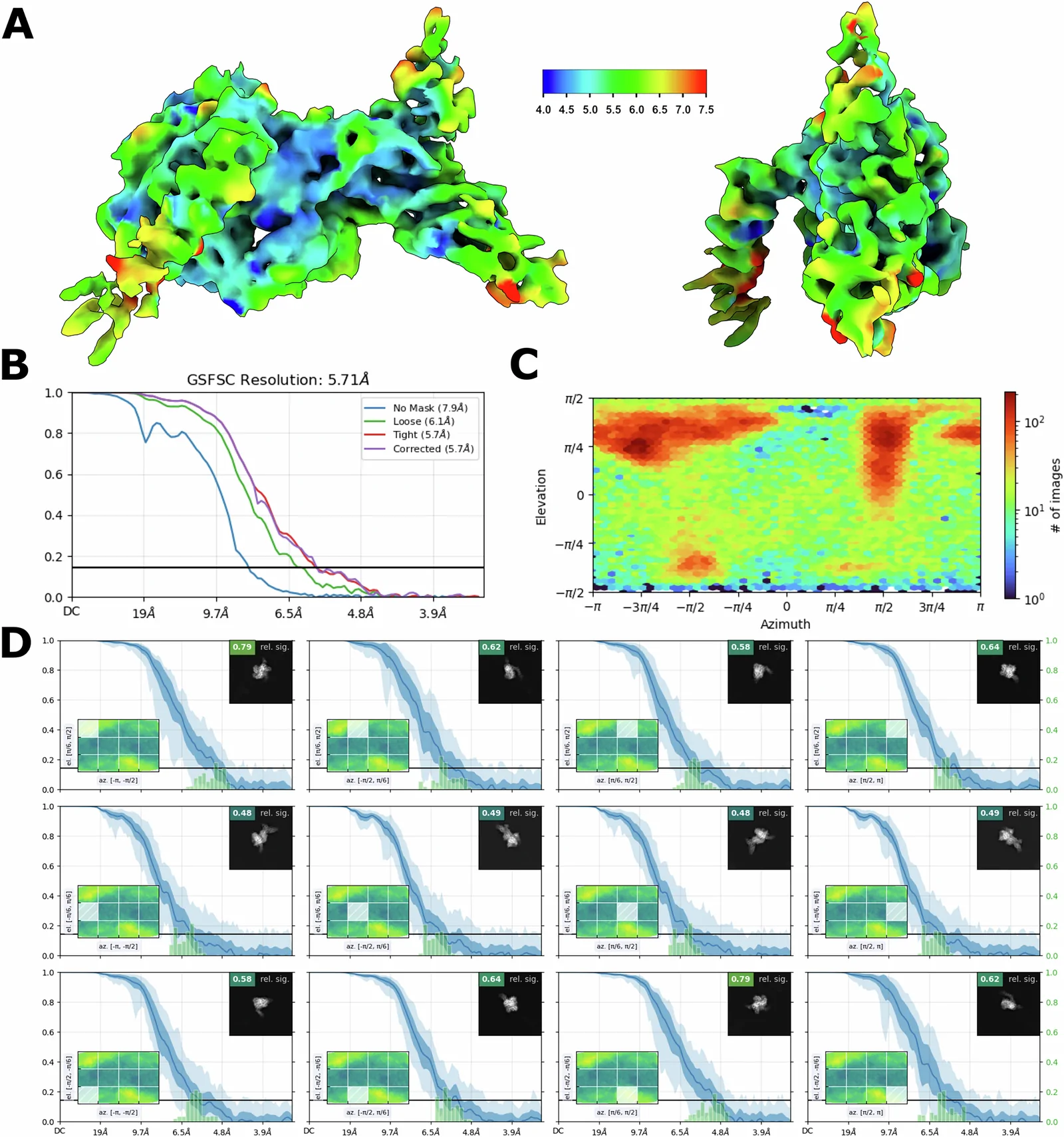
Final Local Refinement CryoEM statistics for OrthoRep Affinity Matured TcdB VHH, VHH*_*TcdB_H2_ortho in complex with TcdB. Local refinement and masking used to reduce noise from His tag and improve resolution. **A**) Local Resolution map (Å), calculated using an FSC value of 0.143 viewed along two different angles. **B**) Global Fourier Shell Correlation plot, Local Refinement. **C**) Orientational distribution plot. **D**) Orientational diagnostics data.

For TcdB, our design campaign targeted the Frizzled-binding epitope located on the RBD. TcdB consists of four functional domains including a central delivery and RBD (DRBD) where the VHHs were designed to bind. Cryo-EM characterization of the original parent design, VHH_TcdB_H2, confirmed that the VHH engages the target Frizzled DRBD epitope (Supplementary Fig. 7). Analysis via two-dimensional and three-dimensional classification revealed a mix of bound and unbound TcdB particles (Fig. 3i and Supplementary Figs. 6 and 7). Extensive three-dimensional classification and local refinement identified multiple structural states of TcdB within the dataset, including an extended bound state (Extended Data Fig. 6 and Supplementary Fig. 8). Three-dimensional refinement of the bound VHH in the extended TcdB state yielded a modest 4.6 Å map, into which the design model was confidently rigid-body docked, showing high agreement with the intended design structure (Fig. 3i–k). To evaluate whether the improved affinity achieved through OrthoRep preserved the original binding mode of the parent design, we performed additional cryo-EM analysis on the affinity-matured VHH, VHH_TcdB_H2_ortho. These experiments revealed a high proportion of TcdB particles now bound by the VHH, consistent with its enhanced affinity (Fig. 3l–n and Supplementary Fig. 3b). Using a similar processing pipeline as for the parent VHH–TcdB complex, we resolved the affinity-matured VHH–TcdB complex to a modest 5.7 Å resolution, enabling us to confidently dock the designed VHH into the cryo-EM density with close agreement. This confirmed that the VHH maintained targeting to the correct epitope and retained its original binding pose after OrthoRep-mediated affinity maturation (Fig. 3l–n and Extended Data Fig. 6). These results underscore the capability of RFdiffusion to design accurate de novo VHHs that are capable of targeting previously unexplored epitopes and are amenable to downstream affinity maturation.

We next used cryo-EM to characterize an affinity-matured VHH (VHH_RBD_D4_ortho19) targeting the SARS-CoV-2 spike RBD, where competition experiments indicated that the parental VHH bound the intended epitope (Fig. 2c, Extended Data Fig. 4c and Supplementary Figs. 3b and 9). The RBD transitions between ‘up’ and ‘down’ conformations, with the ‘up’ state enabling receptor binding and viral entry^32^. Cryo-EM two-dimensional class averages and three-dimensional classification reconstructions of the VHH-bound complex revealed a mixture of RBD conformations (1–2 ‘up’), with VHH density observed exclusively in the up state. This is consistent with its design, as the target epitope is occluded in the down conformation (Supplementary Fig. 9a,b). Global refinement with an average estimated resolution of 3.9 Å provided well-defined density for the lower portion of the spike protein (local resolution of approximately 2.5 Å), but the relative flexibility of the RBD resulted in substantial signal averaging, causing density loss at higher contour levels, which precluded assessment of VHH design accuracy (Supplementary Fig. 9c–e). Symmetry expansion and local refinement helped improve the resolution of the RBD–VHH interface, confirming the intended VHH fold and accurate epitope targeting following rigid-body docking of the design model into the density map (Supplementary Fig. 9f,g), in agreement with our biochemical competition data (Fig. 2c). However, although the VHH bound the correct RBD epitope, its binding mode deviated notably from the design model, instead adopting a predominantly framework-mediated interaction that more closely matched retrospective AlphaFold3 predictions (Supplementary Fig. 9g,h). Owing to the deviation between the designed dock and the experimentally determined dock, we classified this as a design failure.

## Design of scFvs with six designed CDRs

Given the success of RFdiffusion at designing VHHs with three de novo CDRs, we next tested its ability to design both heavy and light chains in scFv format. RFdiffusion was used to generate scFvs targeting specific epitope sites, following a strategy similar to the VHH design approach. However, unlike VHHs, where only three CDRs were built de novo, scFv design involved constructing all six CDRs on both the heavy and the light chains in addition to the docking mode.

**Extended Data Fig. 7:**
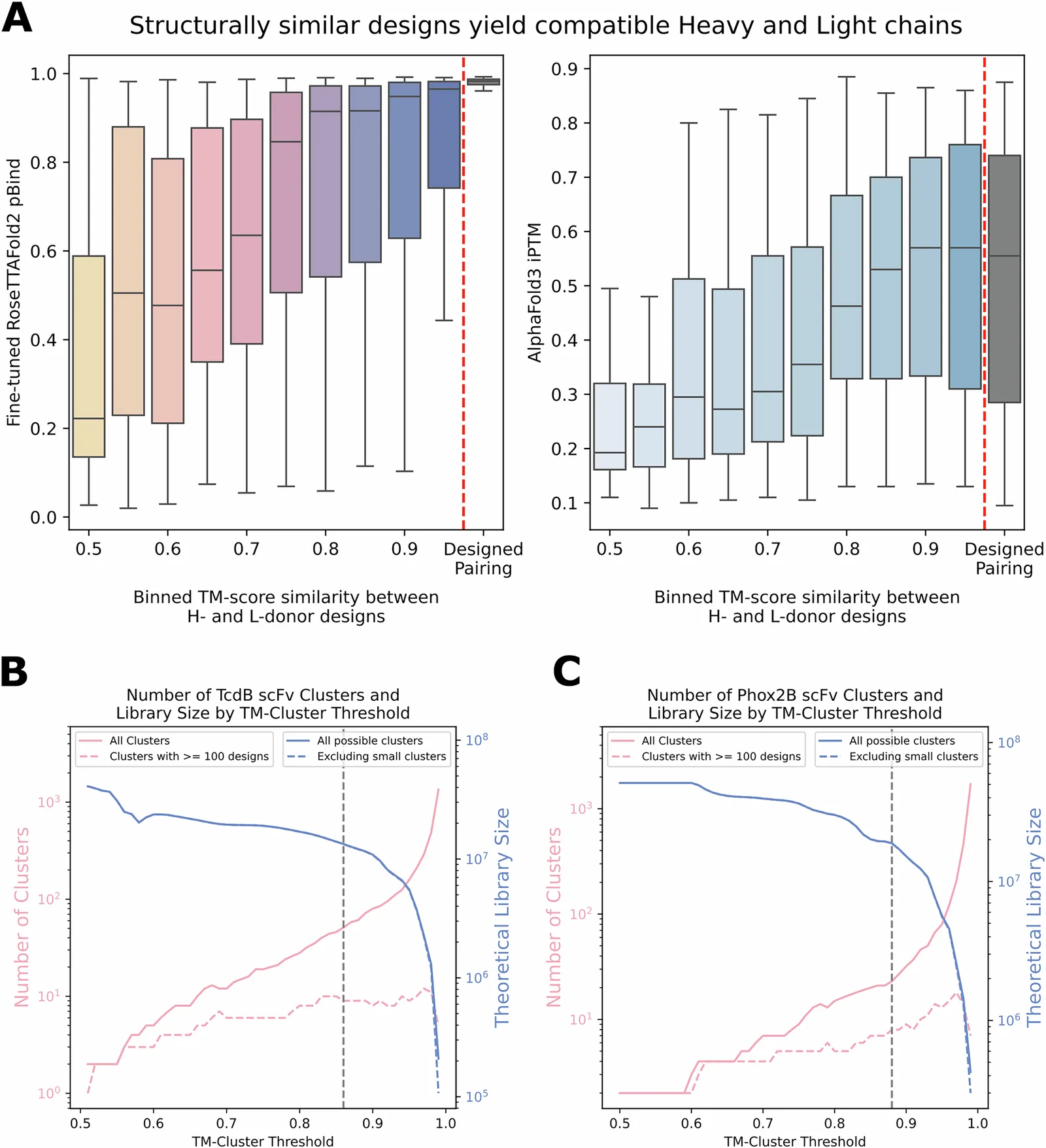
Computational validation of the structure-based combinatorial assembly strategy. Structure-based design permits the rational combinatorial assembly of heavy and light chains, assembling only heavy and light chains from structurally similar pairs. **A**) Fine-tuned RoseTTAFold (left), and AlphaFold3 (right) validate that pairing heavy and light chains from structurally similar (i.e. high pairwise TM score) designs yields scFvs that are more likely to be predicted to bind with high confidence (RF2 pBind, left; AF3 ipTM, right) than heavy and light chains from structurally-dissimilar (low pairwise TM score) designs. Note that the extremely high pBind distribution of the “designed pairings” (rightmost bar of left plot) is an artifact of those designs being specifically filtered for high pBind scores prior to the library being ordered. **B-C**) combinatorial assembly leads to dramatically larger library sizes. Plots show the number of clusters (pink) at different TM score similarity thresholds for TcdB (left) and Phox2b (right) scFvs. For the amplification strategy to work, each “cluster” becomes a PCR subpool, requiring independent PCR reactions (3 per subpool). Hence, we limit ourselves to large subpools (>= 100 designs), which maximizes the combinatorial amplification for the amount of additional library assembly work. We additionally plot the theoretical library size for each target (blue), calculated as *number_of_clusters x cluster_size*^*2*^. Gray lines indicate the TM threshold chosen for library assembly, where library sizes approximately match the transformation efficiency of yeast (10^7^)^52^.

The gene synthesis problem is more formidable for scFvs than for VHHs as they are too long to be simply assembled from pairs of conventional oligonucleotides synthesized on oligonucleotide arrays, and are challenging to uniquely pair due to high sequence homology between scFvs. We developed stepwise assembly protocols that enable the construction of libraries with heavy and light chains either specifically paired as in the design models (Supplementary Figs. 10 and 11) or combinatorially mixed within subsets of designs specifically with similar target-binding modes (Supplementary Fig. 12). The latter approach helps to overcome the greater challenge of accurate design of six CDRs de novo, which increases the possibilities for error compared with the VHH problem as only one suboptimal CDR can compromise binding. We found that given sets of nearly superimposable designs targeting the same site with the same binding mode, new scFvs generated by combining pairs of heavy and light chains from different designs were confidently predicted to bind to the target site in the designed binding mode at similar frequencies as compared to the original designs (Extended Data Fig. 7a). By contrast, random, structure-agnostic pairing rarely led to predicted binders (Extended Data Fig. 7a). Hence, by mixing CDRs from different designs that bind in the same orientation, we can effectively overcome failures due to single imperfectly designed CDRs, thereby offering a combinatorial solution to a combinatorially more complex problem (two-chain scFv design versus one-chain VHH design). This strategy highlights a key advantage of structure-based design: ‘intelligent’ pairing of heavy and light chains is possible with a structural model of every antibody, and allows de novo-designed antibody libraries to reach scales attainable by traditional library assembly methods, despite current limits in gene synthesis.

**Fig. 4:**
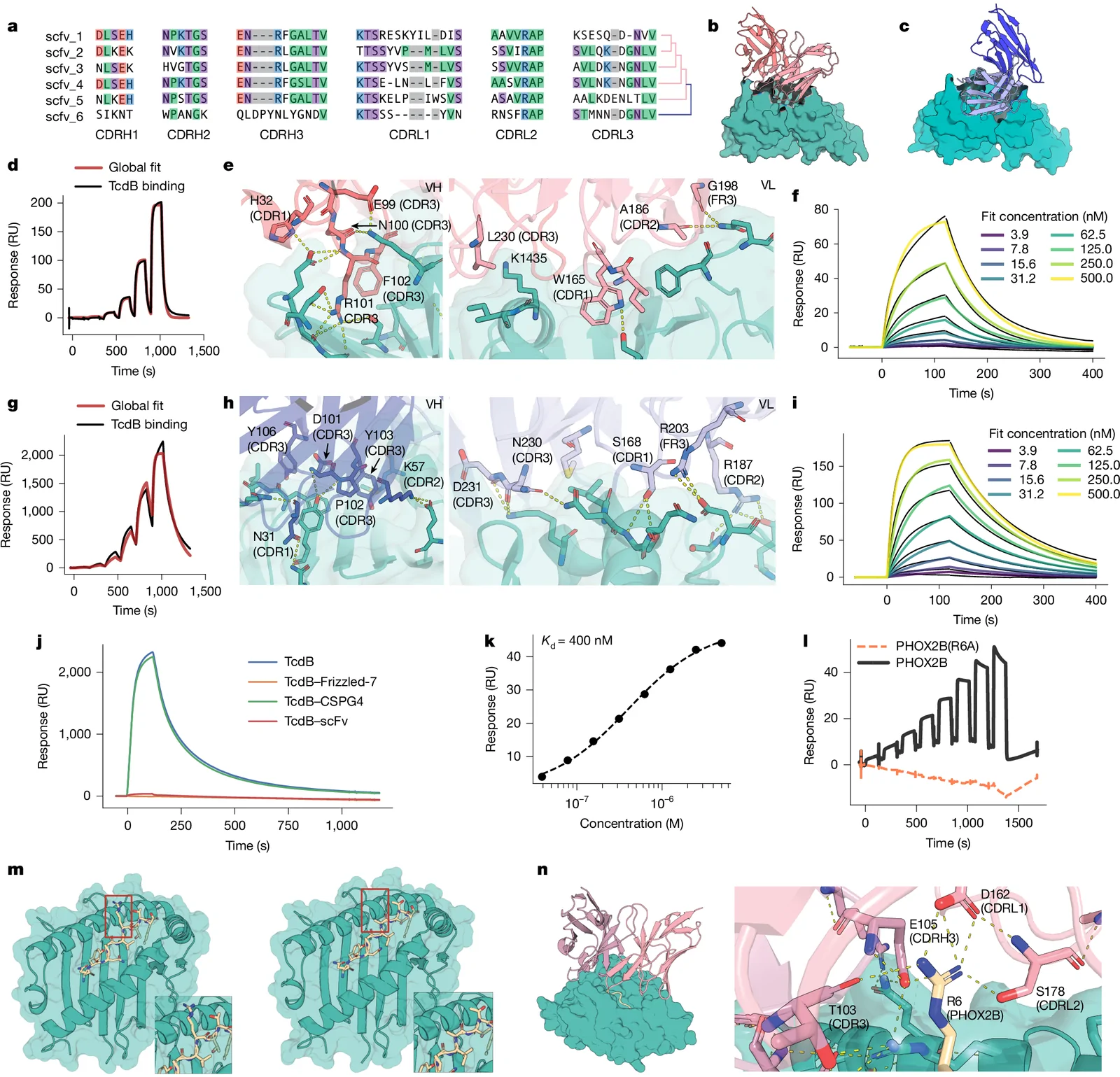
Biochemical characterization of combinatorially assembled scFvs with six designed CDRs. **a**, Multiple sequence alignment of six scFvs that bind to TcdB. scFvs 1–5 originate from the same structural cluster, whereas scFv6 originates from a distinct cluster. **b**,**c**, AlphaFold3 predictions of scFv5 (**b**) and scFv6 (**c**) in complex with TcdB. scFv5 and scFv6 are predicted to bind to a similar but not identical epitope. The predicted orientation of scFv6 relative to TcdB is rotated compared with scFv5. **d**, Affinity of scFv5 to TcdB was 460 nM by SPR. **e**, Computational prediction of the scFv5–TcdB interface for VH (variable heavy-chain fragment; left) and VL (variable light-chain fragment; right). **f**, scFv5, when expressed as a full-length IgG1, shows a binding affinity of 380 nM to TcdB by SPR. **g**, Affinity of scFv6 to TcdB was 72 nM by SPR. **h**, Computational prediction of the scFv6–TcdB interface for VH (left) and VL (right). **i**, scFv6, when expressed as a full-length IgG1, shows a binding affinity of 68 nM to TcdB by SPR. **j**, scFv5 competes with Frizzled-7 and does not compete with CSPG4, indicating on-target binding. scFv5 was conjugated to a CM5 chip and TcdB RBD was flowed over at 50 nM either alone or mixed with 1 μM of Frizzled-7, CSPG4 or scFv5. **k**,**l**, SPR comparative analysis of B1.2.1 binding to C*07:02–PHOX2B versus C*07:02–PHOX2B(R6A). scFv was immobilized and then on-target and off-target binding was measured across an eight-step, twofold titration with an upper concentration of 5 μM. Steady-state kinetic analysis (**k**) and raw SPR trace (**l**) of on-target and off-target binding indicate specific binding to the intended target. **m**, AlphaFold3 predictions of HLA-C*07:02 with peptide PHOX2B (left) and PHOX2B(R6A) (right). R6 of PHOX2B is predicted to be solvent exposed. **n**, AlphaFold3 prediction of scFv B1.2.1 in complex with C*07:02–PHOX2B (left). Predicted polar contacts with R6 of the PHOX2B peptide (right), mediated by CDRH3, CDRL1 and CDRL2, are also shown. Figure was created using BioRender (http://biorender.com).

**Extended Data Fig. 8:**
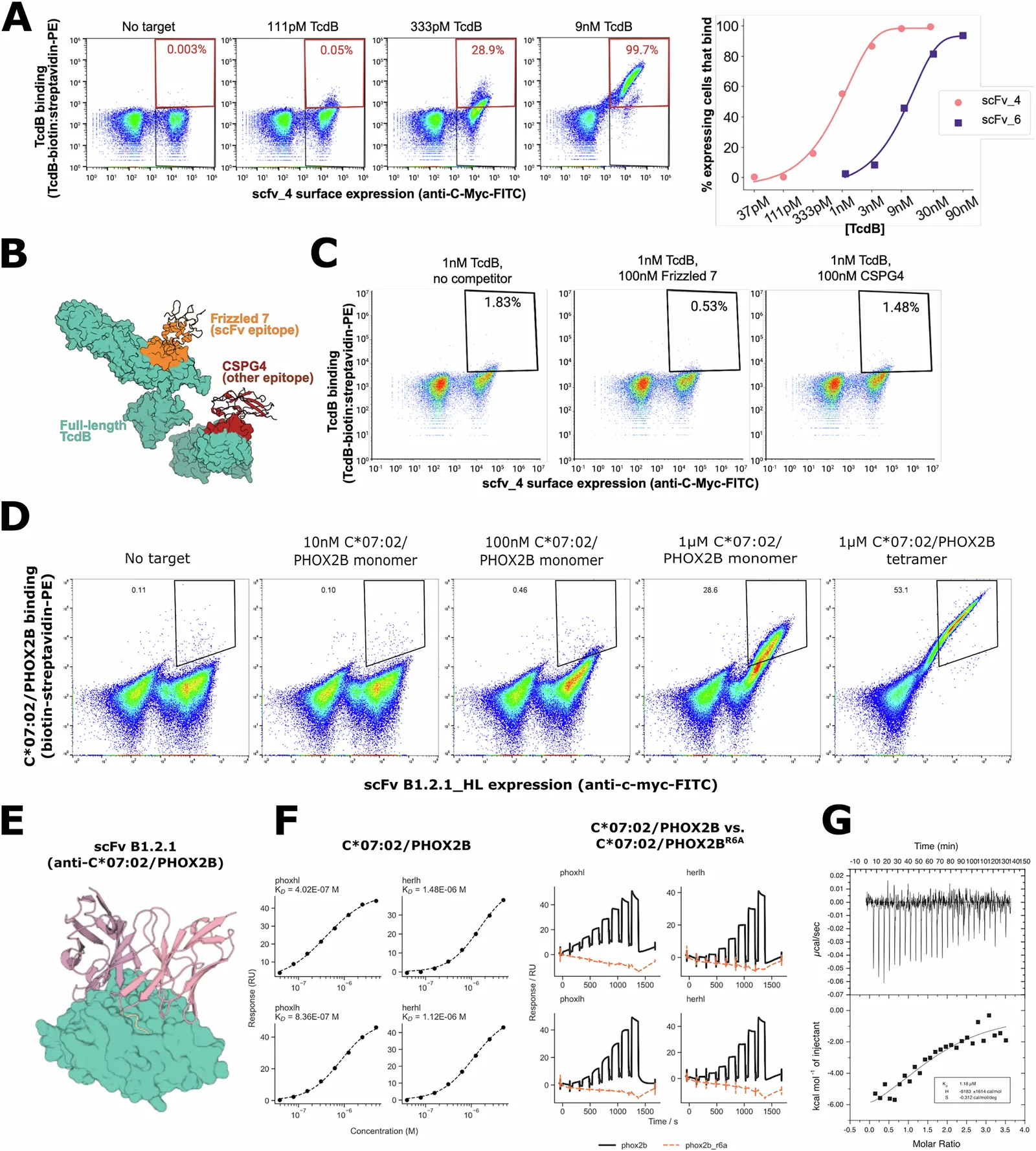
Characterization of TcdB- and Phox2B-binding scFvs. **A-C: TcdB-binding scFvs**. **A**) (left) Results of flow cytometry of yeast samples displaying scFv4-C-Myc construct. Each sample was treated with a titration of soluble biotinylated TcdB fragment (1285-1804) (bn-TcdB) concentrations and visualized with anti-C-Myc FITC + SAPE. (right) Percentage of expressing cells which are within the gate increase with bn-TcdB concentration. **B**) scFvs were designed to bind to the Frizzled epitope and therefore should compete with Frizzled-7. Designed scFvs should not compete with CSPG4, which binds at a different epitope on full-length TcdB. **C**) Yeast displaying scFv4-C-Myc were incubated with 1 nM TcdB and either no competitor, 100 nM Frizzled-7, or 100 nM CSPG4. Binding signal specifically decreases when Frizzled-7 is added, supporting that scFv4 binds at the Frizzled-7 epitope. Binding signal does not significantly decrease when CSPG4 is added. **D-G: Phox2B-binding scFvs**. **D**) C*07:02/PHOX2B titration results with yeast surface display of anti-C*07:02/PHOX2B scFv B1.2.1, tested in the “HL” orientation with a (G4S)3 linker. For the tetramer condition, the biotinylated C:07:02/PHOX2B pHLA was tetramerized on streptavidin-PE (SAPE) prior to validation. The negative control is yeast incubated in the same concentrations of SAPE and FITC used in the experimental conditions in the absence of target. **E**) AF3 prediction of construct B1.2.1 docked to C*07:02/PHOX2B. **F**) Left: Surface plasmon resonance (SPR) data characterizing binding of B1.2.1 in the HL and LH orientations in the 10LH-based framework (“phox” prefix, left column) and the trastuzumab framework (“her” prefix, right column). B1.2.1 binds with approximately 1 μM affinity. Right: SPR data characterizing on-target binding of C*07:02/PHOX2B (“phox2b”) versus the same HLA bound to the R6A mutant of PHOX2B (“phox2b_r6a”). The results indicate specific binding to the intended target. **G**) Representative ITC titration of HLA-C*07:02/PHOX2B (30 μM) into a sample containing 2 μM herceptin_VLVH-His-Avi binder. Both samples contain 1 mM excess of PHOX2B peptide, to prevent the formation of empty HLA. The black line is the fit of the isotherm. Fitted values for KD, ΔH, and ΔS were determined using a 1-site binding model.

We succeeded in identifying epitope-specific scFvs from the heavy–light combinatorial libraries (of a theoretical complexity of approximately 10 million; Extended Data Figs. 7b,c and 8a–c) but not the fixed pairing libraries (Supplementary Fig. 13). Following expression and purification, SPR analysis of six distinct scFvs originating from two unique docks targeting the Frizzled epitope of TcdB revealed a range of affinities (Fig. 4d–h): the highest affinity binder, scFv6, had a *K*_d_ of 72 nM (Fig. 4g). Conversion of the scFv to a full length IgG1 generated antibodies that bind with comparable (68 nM) affinity, demonstrating that our design method can be used to generate full-length antibodies (Fig. 4i). There are no antibodies binding to this epitope in the PDB, hence, this success cannot be attributed to memorization. Subsequent prediction of the structure of the scFv with AlphaFold3 showed a binding mode identical to that of the two nearly superimposable parent designs that contributed the light and heavy chains (Supplementary Fig. 16c,d). Competition with a known receptor, Frizzled-7, to this epitope confirmed that binding of scFv5 was on target (Fig. 4j). By contrast, no competition was seen in the presence of CSPG4, an alternative receptor that interacts with an epitope at the toxin core. Thus, scFvs targeting user-specified epitopes can be identified from structure-aware designed combinatorial libraries.

We next targeted a clinically relevant epitope: the QYNPIRTTF peptide derived from the *PHOX2B* neuroblastoma-dependency gene and master transcriptional regulator in complex with the major histocompatibility complex (MHC) allotype HLA-C*07:02 (we refer to this peptide below simply as PHOX2B). The PHOX2B peptide was originally discovered by immunopeptidomics of neuroblastoma patient-derived samples, and has been targeted with peptide-centric chimeric antigen receptors (PC-CARs) for treating high-risk neuroblastoma^33^. However, the PC-CARs identified previously are restricted to recognizing PHOX2B presented on HLAs of the A9 serological group, excluding the common allotype HLA-C*07:02 (ref. ^34^). Targeting the PHOX2B–HLA-C*07:02 complex could meaningfully increase the addressable patient population for these immunotherapies, and has been the focus of ongoing therapeutics development. Recently, computationally designed (non-antibody) binders for PHOX2B–HLA-C*07:02 have been developed, using the TRACeR-I system^35^, whereas high-affinity TCRs have been identified for targeting peptides on the common HLA-C*08:02/HLA-C*05:01 allotypes^36^. A benefit of structure-based design is the ability to target specific peptide residues to achieve binding specificity (rather than binding only to the MHC), and we therefore used RFdiffusion to target the R6 residue, which is known to be important for binding in the PC-CAR^34^. Given the low stability of the PHOX2B–HLA-C*07:02 complex (*T*_m_ of 44.2 °C)^34^, we leveraged a disulfide-stabilized approach to prepare a stabilized form of the pHLA target^37^. Using the combinatorial assembly approach described above, we identified modest-affinity (400 nM as measured by SPR and 1 μM as measured by isothermal titration calorimetry (ITC); Fig. 4k,l and Extended Data Fig. 8e–g) scFv binders to PHOX2B–HLA-C*07:02. Binding was specific to the peptide, with no detectable binding to the R6A point mutant PHOX2B peptide (PHOX2B(R6A)–HLA-C*07:02; Fig. 4l). Attempts were made to incorporate scFv binders into a 4-1BB-CAR, but T cell cytotoxicity assays demonstrated no detectable killing of a range of neuroblastoma cell lines (Supplementary Fig. 14), probably because of the modest binding affinity and/or low levels of antigen density expressed on the tumour cells. Although there is still considerable room for improvement in affinity, this demonstrates the ability of structure-based antibody design, paired with appropriate library assembly methods, to design specific binders to challenging and clinically important target epitopes.

## Atomically accurate scFv design to TcdB

**Fig. 5:**
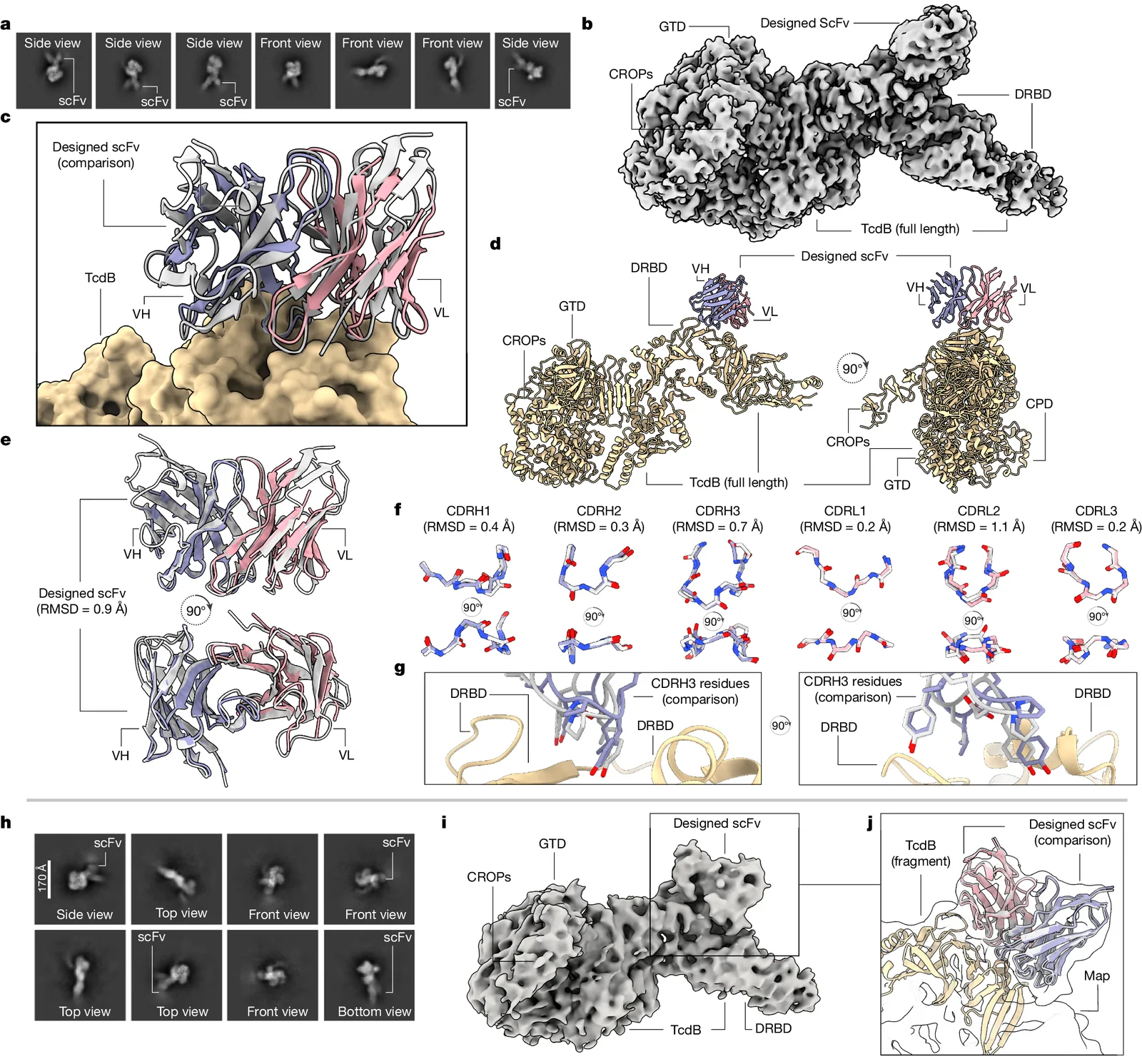
Cryo-EM structural characterization of two TcdB-binding scFvs. **a**, Labelled cryo-EM two-dimensional class averages of a designed scFv, scFv6, bound to TcdB. **b**, A 3.6 Å cryo-EM three-dimensional reconstruction of the complex shows scFv6 bound to TcdB along the Frizzled epitope. **c**, The cryo-EM structure of scFv6 in complex with TcdB closely matches the design model. **d**, Cryo-EM structure of scFv6 bound to TcdB. **e**, Cryo-EM reveals the accurate design of scFv6 using RFdiffusion. **f**, Superposition of each of the six designed scFv6 CDR loop predicted structures as compared with the built cryo-EM structure. **g**, Comparison of predicted CDRH3 rotamers compared with the built 3.6 Å cryo-EM structure. **h**, Labelled cryo-EM two-dimensional class averages of the designed scFv, scFv5, bound to full-length TcdB. **i**, A 6.1 Å cryo-EM three-dimensional reconstruction of the complex shows the scFv5 bound to the target epitope as predicted. **j**, Owing to the modest resolution, a fragment of TcdB was first docked into the cryo-EM density map, and the full design model—including both the TcdB fragment and the designed scFv—was then aligned to the pre-fitted TcdB fragment. This approach demonstrates that the predicted design closely matches the experimentally determined complex in structure, epitope targeting and overall conformation. In models, yellow denotes TcdB; navy indicates the variable heavy-chain fragment (cryo-EM); pink shows the variable light-chain fragment (cryo-EM); and grey denotes the computational design prediction.

**Extended Data Fig. 9:**
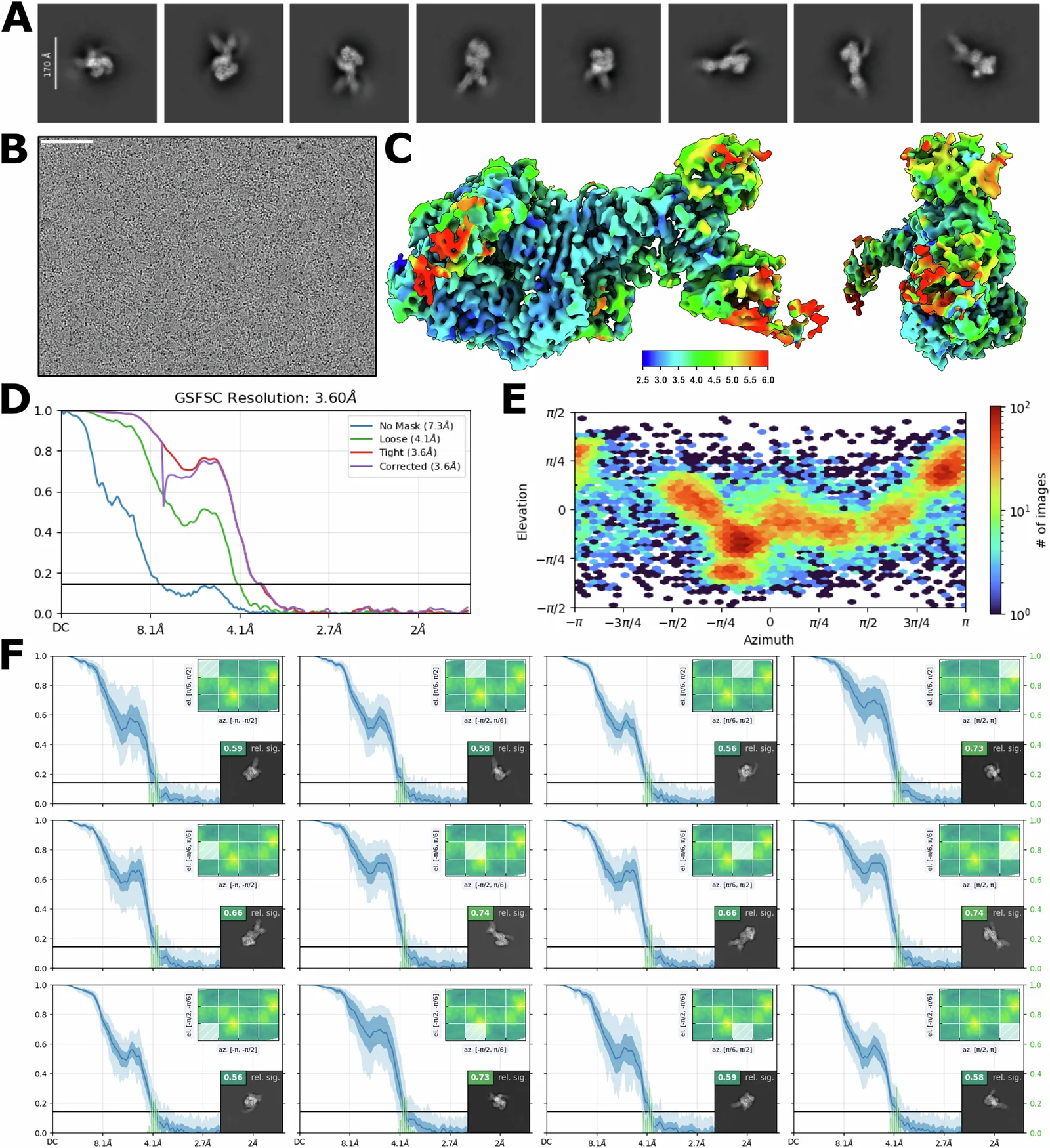
CryoEM statistics for TcdB in complex with *scFv6.* 10,897 movies were collected on a Glacios with a K3 detector. Thin ice was targeted for imaging, and only extended TcdB were observed. Heterogeneous refinement and apo structure were used to sort scFv bound TcdB (41,837 particles) and unbound apo TcdB (14,384 particles). **A**) 2D Class Averages. **B**) Representative micrograph **C**) Local Resolution map (Å), calculated using an FSC value of 0.143 viewed along two different angles **D**) Global Fourier Shell Correlation plot, Non Uniform Refinement **E**) Orientational distribution plots. **F**) Orientational diagnostics data.

To evaluate the accuracy of de novo scFv design, we determined the cryo-EM structures of two combinatorially assembled scFvs, scFv5 and scFv6, both targeting the Frizzled epitope of TcdB. Cryo-EM analysis confirmed that both scFvs bound the Frizzled epitope as designed (Fig. 5 and Extended Data Fig. 9). High-resolution two-dimensional class averages of scFv6 revealed clear density for both TcdB and the bound scFv, further supported by a 3.6 Å three-dimensional reconstruction (Fig. 5a,b and Extended Data Fig. 9). The resolved structure showed that scFv6 engaged the Frizzled epitope along its DRBD domain with the predicted binding orientation (Fig. 5d and Supplementary Methods Table 10). Superposition of the cryo-EM structure with the design model demonstrated remarkable agreement, with both heavy and light chains interacting with the epitope as intended (Fig. 5c and Supplementary Fig. 16a,b). The overall fold closely matched the design, a composite model of the two chains originating from distinct but structurally similar designs (RMSD = 0.9 Å), and each of the six CDRs exhibited near-atomic precision (backbone RMSDs: CDRH1 = 0.4 Å, CDRH2 = 0.3 Å, CDRH3 = 0.7 Å, CDRL1 = 0.2 Å, CDRL2 = 1.1 Å and CDRL3 = 0.2 Å; Fig. 5e,f). This agreement extended to the rotameric conformations of CDR side chains and their interactions with the Frizzled epitope, underscoring the accuracy of RFdiffusion in designing de novo scFv–target interactions (Fig. 5g).

scFv5 was designed to bind to the same epitope but with a distinct approach angle relative to scFv6 (Fig. 4b,c). A 6.1 Å cryo-EM reconstruction confirmed scFv5 binding to the TcdB Frizzled epitope, with two-dimensional class averages showing clear density for the complex (Fig. 5h and Supplementary Fig. 15). Rigid-body docking of the design model into the cryo-EM density revealed close agreement between the predicted-binding and experimentally determined-binding modes (Fig. 5i,j).

## Improved oracles increase success rate

Although our results demonstrate that the de novo design of antibodies is possible, the experimental success rates remain low. A key contributor to previous successes in de novo binder design was improved filters (primarily AlphaFold2 (ref. ^19^), which enriched for experimental success in the subset of designs that are tested experimentally^2,22^. At the outset of this study, we sought to build such a filter by fine-tuning RoseTTAFold2 (Extended Data Figs. 2 and 3), but the filtering power of this model is limited (at least with the settings used; providing 100% of interface ‘hotspots’). This probably accounts for the low experimental success rates and the inaccurate SARS-CoV-2 design, where the overall fold and epitope targeting were correct, but the binding orientation was not.

**Extended Data Fig. 10:**
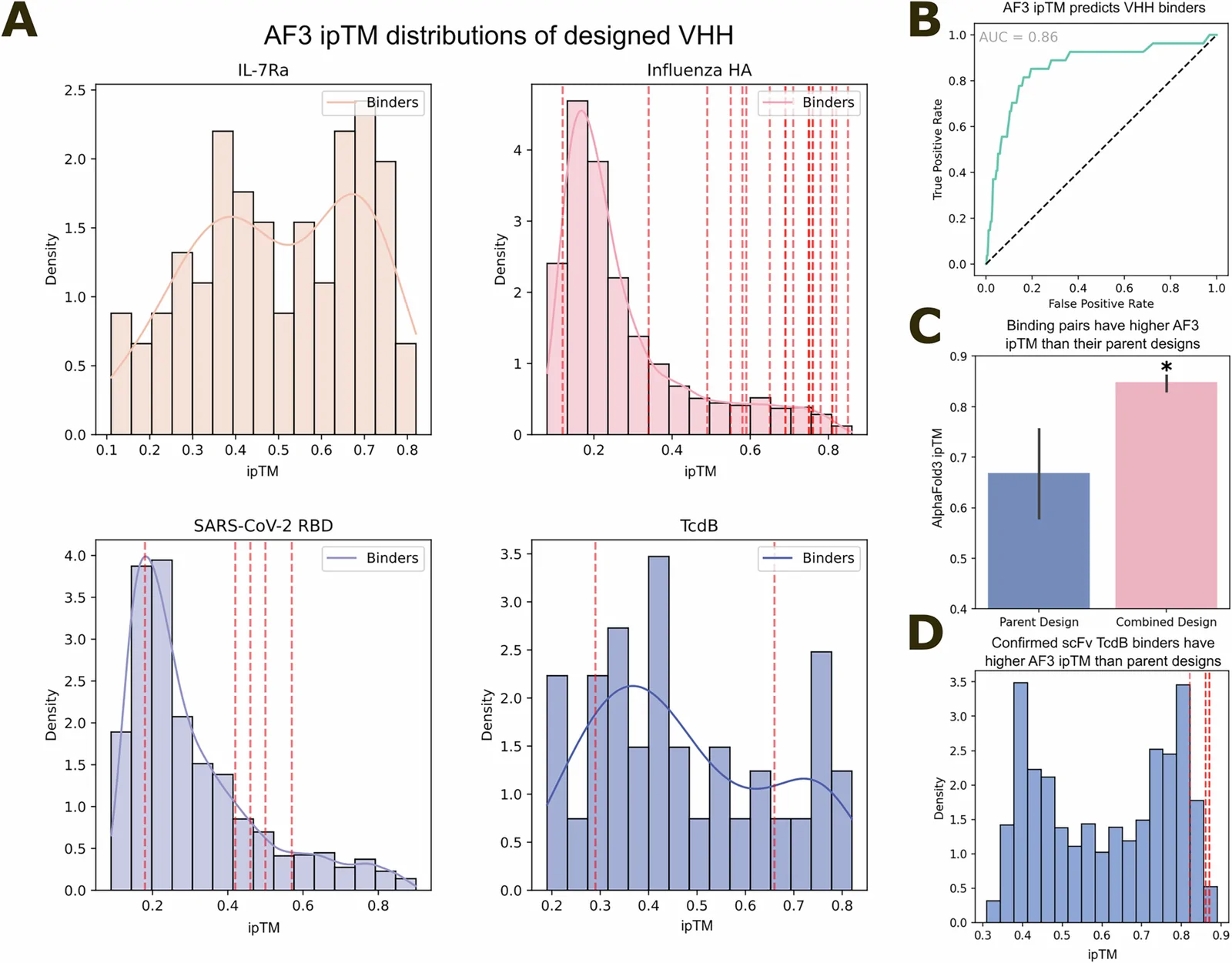
AlphaFold3 retrospectively predicts binders. **A**) ipTM distributions of design VHH libraries against 4 targets. Red lines indicate validated binders. **B**) ROC curve demonstrating strong retrospective predictive power of AF3 at discriminating designed VHH binders from non-binders (AUC = 0.86). Note though that this plot is dominated by influenza HA binders, which are more numerous than confirmed binders to SARS-CoV-2 RBD and TcdB. **C**-**D**) Similar retrospective analyses of TcdB scFv binders. These binders were assembled combinatorially from structurally similar “parent” designs. The successfully-binding combined designs have significantly higher AF3 ipTM scores than the parent designs from which they emanate (**C**, 6 binders from 12 parent designs; two-sided Students t-test; *p* = 0.0025), and from the parental library as a whole (**D**). These analyses indicate the utility of AF3 for antibody design filtering.

Subsequent to the design work in this study, AlphaFold3 (ref. ^24^) was released and has improved antibody structure prediction accuracy^24,38^, both with^24,38^ and without^38^ antigen present. Retrospectively, we can assess how filtering with AlphaFold3 would have improved experimental success rates. First, AlphaFold3 accurately predicts the experimentally validated structure of the inaccurately designed SARS-CoV-2 VHH (Supplementary Fig. 9). Had AlphaFold3 been used as an initial filter, this design would have been rejected due to the discrepancy between the predicted and intended structures, thereby preventing its experimental testing. Second, we predicted the structures of the SARS-CoV-2, influenza haemagglutinin, TcdB and IL-7Rα VHH designs using AlphaFold3 with a multiple sequence alignment (MSA) and templates for the target and only a template for the VHH (as CDRs are de novo, we reasoned the MSA would be of limited utility). We analysed the predictions for libraries with at least one structurally validated VHH (TcdB, influenza haemagglutinin and SARS-CoV-2). These results are dominated by anti-haemagglutinin VHHs as the majority of successful binders came from this library. We found that the AlphaFold3 interface predicted template modelling (ipTM) score, a measure of model confidence over the interface, is predictive of binding success (area under the curve = 0.86; Extended Data Fig. 10a,b). Overall, only 9% of our ordered VHH designs have an ipTM > 0.6, suggesting that success rate will be improved by incorporation of an ipTM filter. We ran a similar analysis for the combinatorially assembled scFv libraries; we predicted the structures of the parental scFv designs (before combinatorial assembly) and the experimentally confirmed scFv designs (combinatorially assembled) using AlphaFold3 with an MSA for the target sequence and templates for the target as well as the heavy and light chains, taking the maximum ipTM score over 10 seeds. We found that successful designs cluster to higher AlphaFold3 ipTM scores than the parental designs (Extended Data Fig. 10c). Only 4% of the initial design library has ipTM > 0.85, whereas 5 out of the 6 experimentally confirmed designs pass this threshold, again suggesting that filtering by AlphaFold3 ipTM should increase success rates (Extended Data Fig. 10d).

## Discussion

Our results demonstrate that de novo design of antibody domains targeting specific epitopes on a target is possible. The cryo-EM structural data for the designed VHHs to influenza haemagglutinin and TcdB reveals very close agreement to the computational design models, showing that our approach can design VHH complexes with atomic accuracy—including the highly variable H3 loop and the overall binding orientation—that are highly dissimilar from any known structures in the PDB. Moreover, cryo-EM structural data of designed scFvs bound to TcdB demonstrate the ability of RFdiffusion to design two-chain scFvs accurately. To our knowledge, these are the first structurally validated cases of de novo-designed antibodies.

Our computational method synergizes with experimental screening approaches developed for retrieving antibodies from large random libraries in several ways. First, yeast display selection methods widely used for antibody library screening enable the retrieval of the highest affinity binders among large sets of designs, which is currently necessary due to the quite low design success rate. Second, screening combinatorial libraries that mix heavy and light chains from designs with similar binding modes allows for the identification of scFvs composed of structurally compatible chains targeting specific epitopes, as demonstrated here for TcdB and PHOX2B–peptide MHC. Third, affinity maturation using OrthoRep^3^ improves the measured affinity of initial VHH designs down to the single-digit nanomolar or subnanomolar range, while preserving the original designed-binding mode. From a practical standpoint, the key advance of this work is not the ability to generate VHHs and scFvs against a target—something often achievable through purely experimental methods—but rather the ability to accurately target specific binding epitopes. The epitope specificity is critical for therapeutic applications such as antagonists that block receptor–ligand interactions, antibodies that avoid competing with endogenous molecules, modulators that induce conformational changes to trigger signalling, or antibodies targeting conserved or evolutionarily restricted viral epitopes.

There remains considerable room for improvement. For the backbone design step, incorporating recent architectural improvements^39^ and new advances in generative modelling^40–42^ may yield design models with higher designability and diversity. RoseTTAFold2 and RFdiffusion have also recently been extended to model all biomolecules (rather than just proteins)^43^, and incorporating this capability into the antibody design RFdiffusion variant should permit the accurate design of antibodies to epitopes containing non-protein atoms, such as glycans. ProteinMPNN was not modified in this current work, but designing sequences that more closely match human CDR sequences would be expected to reduce the potential immunogenicity of designed antibodies^44,45^. Indeed, designed sequences are currently somewhat less human (as assessed by an OASis score^46^) than therapeutic antibody CDRs (Supplementary Fig. 1d). Further improvements in antibody structure prediction methods should allow faster optimization of upstream design methods and improve experimental success rates.

Ultimately, computational de novo design of antibodies using our RFdiffusion and related approaches^47^ could revolutionize antibody discovery and development. As the method improves and success rates increase, it has the potential to be faster and more cost-effective than immunizing animals or screening random libraries. A structure-based approach to antibody design should also aid the optimization of key pharmaceutical properties, such as aggregation, solubility and expression levels (all major challenges in antibody development) in a structure-informed manner. Together, we expect that computational design of antibodies will increase the number of tractable clinical targets and diseases accessible to antibody therapeutics.

### Reporting summary

Further information on research design is available in the Nature Portfolio Reporting Summary linked to this article.

## Online content

Any methods, additional references, Nature Portfolio reporting summaries, source data, extended data, supplementary information, acknowledgements, peer review information; details of author contributions and competing interests; and statements of data and code availability are available at https://doi.org/10.1038/s41586-025-09721-5.

## Supplementary information

Supplementary Information This Supplementary Information file contains tables, figures, and three main text sections: 1) A description of the computational methods used in this study. We describe the fine tuning of RFdiffusion and RF2, and the computational evaluation of these models for antibody design; 2) A description of the experimental methods used to synthesize and characterize the designed antibodies; 3) A description of the electron microscopy methods used to structurally characterize several antibodies in this study.

Reporting Summary

Peer Review File

## Data Availability

The antibody training dataset is available on Zenodo^48^ (https://zenodo.org/records/15741710). Cryo-EM maps and corresponding atomic models are located in the Electron Microscopy Data Bank (EMD-49373 and EMD-49405) and PDB (9NFU and 9NH7).

## Code availability

The codes for running the RFdiffusion antibody design, ProteinMPNN and fine-tuned RoseTTAFold have been released as a single repository on GitHub, free for academic, personal and commercial use (https://github.com/RosettaCommons/RFantibody).
