# Supplementary material for: Atomically accurate de novo design of antibodies with RFdiffusion
Source: Nature. 2025 Nov 5;649(8095):183–93. doi: 10.1038/s41586-025-09721-5 (PMC12727541; doi:10.1038/s41586-025-09721-5)
Supplement: Supplementary file 3 [file 41586_2025_9721_MOESM3_ESM.pdf]

nature portfolio

Peer Review File

Atomically accurate de novo design of antibodies with RFdiffusion

Corresponding Author: Professor David Baker

Any redactions in this file are there to maintain patient confidentiality, the confidentiality of unpublished data, or to remove third-party material.

This file contains all reviewer reports in order by version, followed by all author rebuttals in order by version.

Version 0:

Reviewer comments:

Referee #1

(Remarks to the Author)
The work by Bennett el al is a suite of computational and experimental steps to generate binders (in the form of VHH or scFvs). A critical step is the use of fine-tuned RFdifussion and RoseTTAFold2, although the model also includes ProteinMPNN. The experimental part includes mainly yeast display for selection of best in silico designs and affinity maturation using OrthoRep. There is limited detail of metrics and success rates along the way and thus raises the question on the relative contribution of RFdiffusion to the anatomical accuracy as claimed in the title of the manuscript. The authors acknowledge this by indicating that "While our results demonstrate that the de novo design of antibodies is possible, the quite low experimental success rates currently necessitate the relatively high-throughput screening methods used in this study".
A second conceptual issue is the claim that the main contribution of this work is in the "ability to accurately target specific binding epitopes." This is a valid claim as shown by creating binders to TcdB – but as discussed below, there is ambiguity in what the authors describe as "epitope" – versus the targeting of a 'region' in a target protein.

Main issues
1. There should be a better effort at identifying the contributions to the final designs of the several computational and experimental steps. This may be achieved with an illustrative figure that depicts the steps and outputs of each of the steps, including when applicable the corresponding metrics/number of designs considered as well as experimental outputs such as binding affinities. The only available metrics are: 9000 designs per target; RSV sites I and III, RBD, Influenza HA) or at lower-throughput, 95 designs per target for TcdB, IL-7Rα and influenza HA. How many hits per target were identified?
2. It would have been useful to have for some of the targets/epitopes of a previously characterized mAb or binder to compare the designs vs such ground truth.
3. There is significant experimental support: for example, yeast display screening of designs and then OrthoRep-enabled affinity maturation (the latter based on a method that mimics the natural evolutionary process, based on randomization and antigenic selection). As such, the reader is left wondering about the extent of innovation/improvement over current technologies. In that order, there is no indication of time investment/iteration cycles necessary to generate a possible lead VHH or ScFv. This is important because the authors claim that current methods are "laborious, time-consuming, and can fail to produce antibodies that interact with the therapeutically relevant epitope".
4. The draft indicates; "In all cases, affinity-matured VHHs acquired several mutations relative to the parent designs and improved binding affinities by approximately two orders of magnitude. Thus, when the authors comment "By isolating the bound conformations, we were able to resolve the VHH-TcdB complex to a modest 5.7 Å resolution, enabling us to confidently dock the designed VHH into the cryo-EM density, confirm that the VHH folded into the expected architecture and bound to the target epitope as designed" the reader assumes that the success is also the success of affinity maturation.
5. The work presented here differs from the original ("vanilla") RFdiffusion (and RoseTTAFold2) by fine-tuning on antibodies. Is this sufficient to claim innovation? The text also needs to spell out the contribution of other programs such as ProteinMPNN (now introduced with little detail: "After the RFdiffusion step, we use ProteinMPNN to design the CDR loop sequences") – could not find detail in the Extended Methods file either.
6. There is emphasis on "targeting of any specified epitope on any target of interest". In many cases, knowledge on an epitope implies also knowledge on a paratope, ie., the pre-existence of antibodies already targeting the epitope which could

be subject to improved affinity (as it is done here via OrthoRep) without starting from scratch, from zero. Importantly, throughout the manuscript there is a persistent reference to de novo design based on predefined epitopes. This terminology reflects a conceptual misunderstanding, as epitopes are inherently defined by their interaction with specific antibody paratopes. The more accurate terminology should describe a region, site, or area of the targeted antigen. Antibodies resulting from de novo design will consequently define distinct and unique epitopes within this predefined site, rather than targeting a predetermined epitope that cannot exist independently of an antibody interaction.

7. Design of scFvs seems to be complex as it requires a stepwise assembly protocol to ensure the pairing between heavy and light chains. From the Extended Methods, there is also a screening step through yeast display. What is then the main contributor to success, the new computational framework, or other downstream steps in assembly for the targeting by scFvs of the user-specified epitopes identified from structure-aware designed combinatorial libraries?

8. The manuscript concludes that the method results in anatomically accurate binders. However, while attributed to the computational model, this is the result of a process where a large number of random designs in silico that are screen by HTS assays, affinity matured and then tested by SPR for binding and specificity, and then the best binder tested by cryoEM. How do the experimental steps contribute to the anatomical accuracy?

9. The mode of binding of VHH_flu_01 to the HA stem region would merit a more detailed comparison with that of naturally isolated mAbs (such as CR6261 shown in Extended Data Fig. GH) that have shown common structural features in the interaction with the stem region, particularly with the W21 aromatic pocket (e.g., F54 and Y98 in most VH1-69 anti-stem antibodies).

10. Which is the degree of humanness of all the generated antibodies? this is a relevant aspect for their developability, as mentioned in the Discussion.

11. Other points:

Discussion: would be good to comment on other approaches to solve the design of antibodies: example
https://pubmed.ncbi.nlm.nih.gov/39793083/, https://www.biorxiv.org/content/10.1101/2024.11.11.622872v1,
https://arxiv.org/abs/2306.12360
https://arxiv.org/abs/2409.08022

Figures: Fig 1 – there is an implicit assumption that Rediffusion needs a target structure and epitope for design. Please discuss.

Minor

1. As indicated above, the manuscript would benefit from a schematic figure presenting the steps of the VHH or scFvs design (computing and experimental steps)

2. “Instead, antibody discovery currently relies on animal immunization or random library screening approaches.” - this sentence omits the frequent identification of antibodies from human memory B cells or plasma cells which are recognized as the optimal source for the isolation of anti-infective antibodies.

3. The directed evolution platform OrthoRep is not defined nor referenced in the abstract (https://www.nature.com/articles/s41467-020-19539-6)

4. The following comment in Introduction seems counterintuitive, as it seems to indicate that it is positive that there are few antibody sequences in PDB : “RoseTTAFold2 (RF2) and RFdiffusion are trained on the entire Protein Data Bank (PDB16) which helps overcome the problem that the PDB contains relatively few antibody structures (~8,100 antibody structures versus >200,000 total structures), complicating the training of large machine learning models.

5. The authors hint at how AlphaFold3 would fit in the current protocol. In particular because of the comment that “A key contributor to previous successes in de novo binder design was improved filters (primarily AlphaFold244), which enriched for experimental success in the subset of designs that are tested experimentally9,20. At the outset of this study, we sought to build such a filter by fine-tuning RoseTTAFold2 (Extended Data Figs. 2-4), but the filtering power of this model is limited”. It would be useful to have a comment regarding the results of Hitawala and Gray (ref 45).

6. The manuscript indicated that “The CDR loops are distinct from VHHs observed in nature, indicating significant generalization beyond the training dataset” – an alternative statement is that CDR diversity is just reflecting hallucination. The statement would be stronger if referring to successful binders.

7. What is the size of libraries in steps such as: “We succeeded in identifying epitope specific scFvs from the heavy-light combinatorial libraries”.

8. The authors make a passing comment in Discussion that “A structure-based approach to antibody design enables the optimization of key pharmaceutical properties, such as aggregation, solubility, and expression levels, in a structurally informed manner.” This risks to be interpreted as an easy and logical task – but it is far from simple.

9. It is not very clear from the text which is the “intended epitope” against which the RBD VHH were generated against.

10. In Extended Data Figure 5 E and H the provided PBD code (6YFT) is wrong (it is referred to the VLP of Wenzhou levi-like virus 1).

11. In Extended Data Figure 8, the order of the panels should be grouped by targeted antigen. The current order s

(Remarks on code availability)

Referee #2

(Remarks to the Author)
I co-reviewed this manuscript with one of the reviewers who provided the listed reports.

(Remarks on code availability)
The work by Bennett el al is a suite of computational and experimental steps to generate binders (in the form of VHH or scFvs). A critical step is the use of fine-tuned RFdifussion and RoseTTAFold2, although the model also includes ProteinMPNN. The experimental part includes mainly yeast display for selection of best in silico designs and affinity

maturation using OrthoRep. There is limited detail of metrics and success rates along the way and thus raises the question on the relative contribution of RFdiffusion to the anatomical accuracy as claimed in the title of the manuscript. The authors acknowledge this by indicating that "While our results demonstrate that the de novo design of antibodies is possible, the quite low experimental success rates currently necessitate the relatively high-throughput screening methods used in this study".
A second conceptual issue is the claim that the main contribution of this work is in the "ability to accurately target specific binding epitopes." This is a valid claim as shown by creating binders to TcdB – but as discussed below, there is ambiguity in what the authors describe as "epitope" – versus the targeting of a 'region' in a target protein.

Main issues
1. There should be a better effort at identifying the contributions to the final designs of the several computational and experimental steps. This may be achieved with an illustrative figure that depicts the steps and outputs of each of the steps, including when applicable the corresponding metrics/number of designs considered as well as experimental outputs such as binding affinities. The only available metrics are: 9000 designs per target; RSV sites I and III, RBD, Influenza HA) or at lower-throughput, 95 designs per target for TcdB, IL-7Rα and influenza HA. How many hits per target were identified?
2. It would have been useful to have for some of the targets/epitopes of a previously characterized mAb or binder to compare the designs vs such ground truth.
3. There is significant experimental support: for example, yeast display screening of designs and then OrthoRep-enabled affinity maturation (the latter based on a method that mimics the natural evolutionary process, based on randomization and antigenic selection). As such, the reader is left wondering about the extent of innovation/improvement over current technologies. In that order, there is no indication of time investment/iteration cycles necessary to generate a possible lead VHH or ScFv. This is important because the authors claim that current methods are "laborious, time-consuming, and can fail to produce antibodies that interact with the therapeutically relevant epitope".
4. The draft indicates; "In all cases, affinity-matured VHHs acquired several mutations relative to the parent designs and improved binding affinities by approximately two orders of magnitude. Thus, when the authors comment "By isolating the bound conformations, we were able to resolve the VHH-TcdB complex to a modest 5.7 Å resolution, enabling us to confidently dock the designed VHH into the cryo-EM density, confirm that the VHH folded into the expected architecture and bound to the target epitope as designed" the reader assumes that the success is also the success of affinity maturation.
5. The work presented here differs from the original ("vanilla") RFdiffusion (and RoseTTAFold2) by fine-tuning on antibodies. Is this sufficient to claim innovation? The text also needs to spell out the contribution of other programs such as ProteinMPNN (now introduced with little detail: "After the RFdiffusion step, we use ProteinMPNN to design the CDR loop sequences") – could not find detail in the Extended Methods file either.
6. There is emphasis on "targeting of any specified epitope on any target of interest". In many cases, knowledge on an epitope implies also knowledge on a paratope, ie., the pre-existence of antibodies already targeting the epitope which could be subject to improved affinity (as it is done here via OrthoRep) without starting from scratch, from zero. Importantly, throughout the manuscript there is a persistent reference to de novo design based on predefined epitopes. This terminology reflects a conceptual misunderstanding, as epitopes are inherently defined by their interaction with specific antibody paratopes. The more accurate terminology should describe a region, site, or area of the targeted antigen. Antibodies resulting from de novo design will consequently define distinct and unique epitopes within this predefined site, rather than targeting a predetermined epitope that cannot exist independently of an antibody interaction.
7. Design of scFvs seems to be complex as it requires a stepwise assembly protocol to ensure the pairing between heavy and light chains. From the Extended Methods, there is also a screening step through yeast display. What is then the main contributor to success, the new computational framework, or other downstream steps in assembly for the targeting by scFvs of the user-specified epitopes identified from structure-aware designed combinatorial libraries?
8. The manuscript concludes that the method results in anatomically accurate binders. However, while attributed to the computational model, this is the result of a process where a large number of random designs in silico that are screen by HTS assays, affinity matured and then tested by SPR for binding and specificity, and then the best binder tested by cryoEM. How do the experimental steps contribute to the anatomical accuracy?
9. The mode of binding of VHH_flu_01 to the HA stem region would merit a more detailed comparison with that of naturally isolated mAbs (such as CR6261 shown in Extended Data Fig. GH) that have shown common structural features in the interaction with the stem region, particularly with the W21 aromatic pocket (e.g., F54 and Y98 in most VH1-69 anti-stem antibodies).
10. Which is the degree of humanness of all the generated antibodies? this is a relevant aspect for their developability, as mentioned in the Discussion.
11. Other points:
Discussion: would be good to comment on other approaches to solve the design of antibodies: example
https://pubmed.ncbi.nlm.nih.gov/39793083/, https://www.biorxiv.org/content/10.1101/2024.11.11.622872v1,
https://arxiv.org/abs/2306.12360
https://arxiv.org/abs/2409.08022
Figures: Fig 1 – there is an implicit assumption that Rediffusion needs a target structure and epitope for design. Please discuss.
Minor
1. As indicated above, the manuscript would benefit from a schematic figure presenting the steps of the VHH or scFvs design (computing and experimental steps)
2. "Instead, antibody discovery currently relies on animal immunization or random library screening approaches." - this sentence omits the frequent identification of antibodies from human memory B cells or plasma cells which are recognized as the optimal source for the isolation of anti-infective antibodies.
3. The directed evolution platform OrthoRep is not defined nor referenced in the abstract
(https://www.nature.com/articles/s41467-020-19539-6)

4. The following comment in Introduction seems counterintuitive, as it seems to indicate that it is positive that there are few antibody sequences in PDB : “RoseTTAFold2 (RF2) and RFdiffusion are trained on the entire Protein Data Bank (PDB16) which helps overcome the problem that the PDB contains relatively few antibody structures (~8,100 antibody structures versus >200,000 total structures), complicating the training of large machine learning models.
5. The authors hint at how AlphaFold3 would fit in the current protocol. In particular because of the comment that “A key contributor to previous successes in de novo binder design was improved filters (primarily AlphaFold244), which enriched for experimental success in the subset of designs that are tested experimentally9,20. At the outset of this study, we sought to build such a filter by fine-tuning RoseTTAFold2 (Extended Data Figs. 2-4), but the filtering power of this model is limited”. It would be useful to have a comment regarding the results of Hitawala and Gray (ref 45).
6. The manuscript indicated that “The CDR loops are distinct from VHHs observed in nature, indicating significant generalization beyond the training dataset” – an alternative statement is that CDR diversity is just reflecting hallucination. The statement would be stronger if referring to successful binders.
7. What is the size of libraries in steps such as: “We succeeded in identifying epitope specific scFvs from the heavy-light combinatorial libraries”.
8. The authors make a passing comment in Discussion that “A structure-based approach to antibody design enables the optimization of key pharmaceutical properties, such as aggregation, solubility, and expression levels, in a structurally informed manner.” This risks to be interpreted as an easy and logical task – but it is far from simple.
9. It is not very clear from the text which is the “intended epitope” against which the RBD VHH were generated against.
10. In Extended Data Figure 5 E and H the provided PBD code (6YFT) is wrong (it is referred to the VLP of Wenzhou levi-like virus 1).
11. In Extended Data Figure 8, the order of the panels should be grouped by targeted antigen. The current order seems random.

Referee #3

(Remarks to the Author)
The paper by Bennett, Watson et al. addresses one of the most relevant questions in modern computational protein design: the development of methods to design antibodies in silico that exhibit high potency and bind to specific epitopes. The authors develop a fine-tuned version of their early tool, RFDiffusion, to generate de novo antibodies with a diffusion protocol; and a fine-tuned version of their also earlier tool, RoseTTAFold 2, which is used to select promising designs with high success. The authors use these tools to design single-domain antibodies against various proteins, and later also scFvs. The antibodies designed by the authors exhibit potencies in the double- and triple-digit nanomolar range, which can generally be optimized by standard affinity maturation to a level of affinity that enables structural characterization by CryoEM. Indeed, the experimental structures show, in most cases, an uncanny resemblance to the designs. The exposition of results is quite convincing, and I think it is fair to state that the authors have indeed done “atomically accurate de novo design of antibodies” as claimed by the title, in what would be the first achievement of its kind.

This work is one of the most interesting articles I have ever read, considering one of the most relevant problems in computational protein design with a clear translational application into antibody drug discovery. The contribution to the literature is obvious and very significant, and I expect broad interest from those working in antibody drug discovery, but also in protein science, in general. The article is technically sound and well-written, and in my opinion more than worthy of publication at Nature. I am delighted to recommend that the article is published.

I would however like to request that the authors address a few questions before the article is fully accepted for publication:

- In the abstract, the authors state that initial computational designs exhibit “modest” affinity — could I suggest that they specify the actual affinity levels i.e. around triple-digit nanomolar?
- The final section constitutes, in my opinion, one of the most thought-provoking parts of the paper, in particular the authors' claim that, had AlphaFold 3 been available, the success rate of experimental designs would have been notably increased. I think this raises the interesting question of exactly how good the fine-tuned version of RoseTTAFold 2 is at predicting and filtering antibody structures. Would the authors be able to compare Extended Figures 3 and 4 to un-tuned RF2, and naïve AF2 and AF3?
- The comparison in Extended Figure 3 is only done against IgFold, which is one of the available methods and not always the most accurate. Would the authors be able to also compare against IgFold? I would also appreciate if the figures could report average RMSDs of both methods, rather than just Wilcoxon tests.
- Why does fine-tuned RF2 work so well on antibody structures? The prevailing view about antibody structure prediction has been that, since deep learning models rely on processing the MSA for evolutionary contact information and implicitly projecting it into 3D space (see e.g. the OpenFold paper by M. AlQuraishi, or the papers by S. Ovchinnikov), and antibodies do not have an evolutionary story, predicting CDR structures is hard. The authors seem to solve the problem by fine-tuning on a specific database (SAbDab + TCR structures + mined loop-target structures from the PDB), and a set of negative examples (mismatching targets + swapping CDRs + an auxiliary yeast display test set). Intuitively, I would have argued that the first approach would have little impact because the structures should already be in the training set of RF2/AF2/AF3, and that mismatching targets or swapping CDRs would have a limited example. Would the authors be able to provide some ablation study (even on just a couple of epochs, to see tendencies) of how much each of these approaches contribute to the significant improvement in RF2?
- On that note, could the authors provide more information about the yeast display data they use in model fine-tuning (section 5.12 of the extended data)? How much data was generated, and how relevant is this for the final performance of the model?
- Finally, I would ask if the authors could report an estimate of the GPU resources required to finetune RF2 and RFdiffusion, so as to estimate the amount of FLOPs required for future research.

On a side comment, the code provided by the authors in the submission appears essentially identical to the public GitHub repository for RFdiffusion 1.1, rather than the RFantibody repository -- was this intended? I would ask if the authors are planning to release any code on the training code used to fine-tune the base models.

I would like to conclude by commending the authors for the extraordinary achievements reported here, and for their commitment to open science by disseminating them in this article.

Carlos Outeiral

(Remarks on code availability)
The code provided by the authors appears essentially identical to the public GitHub repository for RFdiffusion 1.1, rather than the RFantibody repository.

Referee #4

(Remarks to the Author)
In this study, the authors report de novo antibody design using a fine-tuned RFdiffusion model combined with experimental screening and optimization. The authors demonstrated the ability to generate functional VHHs and scFvs targeting disease-relevant epitopes. Several of the designs were confirmed with cryo-EM structures. The biological relevance of the case studies is compelling, particularly the targeting of the conserved HA stem epitope and the Frizzled-7 interface of TcdB, which lacks existing antibody structures. The study clearly represents a significant advance in computational antibody design.

However, several issues need to be addressed to better reflect the pipeline's strengths and limitations, enhance its reproducibility, and accelerate adoption by relevant communities.

1.The current manuscript lacks a figure that visually summarizes the overall computational and experimental pipeline, requiring readers to piece together the steps from the text through which the authors achieved their final goals. The current Figure 1 focuses only on the computational aspects and does not adequately represent the broader workflow. Furthermore, panels B to D of Figure 1 are highly repetitive, and the straightforward progression between them has already been clearly presented in the main text. It can be very helpful to replace these panels with a multi-panel schematic figure to outline the key steps, from epitope-targeted CDR design to experimental screening and affinity maturation.

2.Additional tables or plots are needed to highlight important results regarding the intrinsic accuracy and success rate of the design protocols, as well as the efficiency of the computational and experimental filtering strategies. Example data to include in these tables might consist of: the number of initial RFdiffusion designs per target, the fraction retained after RF2 filtering (with pAE/iPTM thresholds), hit rates in experimental screening, affinity maturation success rates (e.g., fold-improvement), etc..

3. The fine-tuning of RFdiffusion and RoseTTAFold2 is central to this work. However, the currently released repository lacks the training/fine-tuning code and datasets. Releasing these resources, especially the datasets, can enhance the reproducibility of the study and facilitate benchmarking against alternative methods.

(Remarks on code availability)
See comments to authors.

Referee #5

(Remarks to the Author)
I co-reviewed this manuscript with one of the reviewers who provided the listed reports.

(Remarks on code availability)

Version 1:

Reviewer comments:

Referee #1

(Remarks to the Author)
I thank the author for bringing significant clarity to key aspects of the work: de novo vs optimization, success rates, epitope/paratope description, release of alphafold3, humanness and critically, the emphasis that atomically accuracy is derived from the design and not from the ulterior steps of affinity optimization.

(Remarks on code availability)

Referee #2

(Remarks to the Author)
I co-reviewed this manuscript with one of the reviewers who provided the listed reports.

(Remarks on code availability)

Referee #3

(Remarks to the Author)
I am satisfied by the authors' remarks on my comments, and the comments of the other reviewers. I am delighted to recommend the article for publication.

Carlos Outeiral

(Remarks on code availability)
The code is, for the most part, clean and well-documented.

Referee #4

(Remarks to the Author)
The authors have systematically addressed the reviewers' suggestions and made effective revisions. The updated manuscript objectively reflects the current advances in the field of computational antibody design. Overall, it represents a timely and significant contribution.

(Remarks on code availability)

Open Access This Peer Review File is licensed under a Creative Commons Attribution 4.0 International License, which permits use, sharing, adaptation, distribution and reproduction in any medium or format, as long as you give appropriate credit to the original author(s) and the source, provide a link to the Creative Commons license, and indicate if changes were made.
In cases where reviewers are anonymous, credit should be given to 'Anonymous Referee' and the source.
The images or other third party material in this Peer Review File are included in the article's Creative Commons license, unless indicated otherwise in a credit line to the material. If material is not included in the article's Creative Commons license and your intended use is not permitted by statutory regulation or exceeds the permitted use, you will need to obtain permission directly from the copyright holder.
To view a copy of this license, visit https://creativecommons.org/licenses/by/4.0/

Referee #1 (Remarks to the Author):

We sincerely thank the two co-reviewers for their very in-depth review of the manuscript, which we believe is fair and balanced, and highlights some mistakes in the submission, as well as some excellent suggestions of experiments that we are happy to include in our revised manuscript. We thank the reviewers for their contribution to this work!

To begin with, we wish to make a distinction, which we had tried to make clear in the original manuscript, between **de novo design** of antibodies and **optimization** of antibodies.

De novo design means "from nothing". In other words, we do not start from a known antibody sequence/structure. The problem can be stated as:

"Given a specific binding site on a target protein, design an antibody that binds to this binding site".

This is the problem we address, for the first time, in this work.

Antibody **optimization**, on the other hand, starts from a *known* antibody, and seeks to optimize it, typically for binding affinity, but also for downstream developability. This is a very extensive field, which we do not seek to contribute to in this work. This problem can be stated as:

"Given a known antibody that binds to a known epitope, optimize that antibody for a given property".

In this work, we are only concerned with the **de novo** problem.

We try to make clear in the manuscript that this work is the **first** demonstration of de novo antibody design, *not* the **final** demonstration. We are honest and upfront about the capabilities of our pipeline:

- *"quite low experimental success rates"*
- *"computational designs exhibit modest affinity"*
- *"Due to the significant deviation between the designed docks and the experimentally-determined dock, we classified this as a design failure."*
- *"filtering by AF3 [AlphaFold3] iPTM should increase success rates"*

The point of this manuscript is that:

- De novo, atomically-accurate antibody design is now *possible*
- To lay the foundations of *how* the field is going to improve upon this work.

Because the described pipeline is not always able to generate very high affinity nanobodies/antibodies (which we did not claim), some affinity optimization of designs (with OrthoRep) was required to determine the structural accuracy of the designs. This was the case for the SARS-CoV-2 VHH and the TcdB VHH (but not for the Influenza HA VHH or TcdB scFvs; note also that we do show with low-resolution cryo-EM that the

non-affinity-matured TcdB VHH was structurally accurate; new Fig. 3I-K). We want to highlight to the reviewers, however, that this affinity maturation was *only* so that we could determine the structures of these VHH. The design structure of the VHHs (and thereby the epitope and paratope) was designed by our **de novo** design pipeline; these are unaffected by the affinity maturation step (Fig. 3I-K). In other words, to show that the designed structure was correct, we used affinity maturation to confirm the binding mode.

We hope that this preamble to our response helps to address many of the reviewers' points below.

To help make the distinction between **de novo** design and antibody **optimization** clearer for the reader, we have significantly reworked the introduction to make this distinction easier for the reader.

We now respond to each comment in turn below.

> The work by Bennett el al is a suite of computational and experimental steps to generate binders (in the form of VHH or scFvs). A critical step is the use of fine-tuned RFdifussion and RoseTTAFold2, although the model also includes ProteinMPNN. The experimental part includes mainly yeast display for selection of best in silico designs and affinity maturation using OrthoRep.
>
> There is limited detail of metrics and success rates along the way and thus raises the question on the relative contribution of RFdiffusion to the anatomical accuracy as claimed in the title of the manuscript. The authors acknowledge this by indicating that "While our results demonstrate that the de novo design of antibodies is possible, the quite low experimental success rates currently necessitate the relatively high-throughput screening methods used in this study".
>
> We have included an additional table (Supplementary Methods Table 6) that summarizes each screen for the targets tested, the method used to determine binding initially and the number of successful designs. Because some were screened immediately as soluble designs through SPR (TcdB, IL7Ra) and others initially through yeast display (RSV F protein, influenza, SARS-CoV-2), we do not report a global success rate as these methods have different sensitivity. We instead report success rates for each experiment to provide a comprehensive overview. Overall, this table aligns with the claim that success rates were "modest".
>
> A second conceptual issue is the claim that the main contribution of this work is in the "ability to accurately target specific binding epitopes." This is a valid claim as shown by creating binders to TcdB – but as discussed below, there is ambiguity in what the authors describe as "epitope" – versus the targeting of a 'region' in a target protein.
>
> We address this point in more detail below, but we agree it is a somewhat imprecise use of the word "epitope". We do show that after fine-tuning,

RFdiffusion does very reliably design VHH/scFvs that do interact with the residues/epitope/region specified by the user, however (Extended Data Fig. 1B).

Main issues

1. There should be a better effort at identifying the contributions to the final designs of the several computational and experimental steps. This may be achieved with an illustrative figure that depicts the steps and outputs of each of the steps, including when applicable the corresponding metrics/number of designs considered as well as experimental outputs such as binding affinities. The only available metrics are: 9000 designs per target; RSV sites I and III, RBD, Influenza HA) or at lower-throughput, 95 designs per target for TcdB, IL-7Rα and influenza HA. How many hits per target were identified?

Supplementary Methods Table 6 now includes the hits per target as well as summary statistics for each screen. We have updated Figure 1 to explain the whole pipeline which includes both the computational and experimental steps. We thank the reviewer for this nice suggestion, which indeed adds clarity!

2. It would have been useful to have for some of the targets/epitopes of a previously characterized mAb or binder to compare the designs vs such ground truth.

We thank the reviewer for this suggestion. We infer two possible interpretations of what is being suggested here:

a.) For targets/epitopes where there is a VHH/antibody in the PDB (the training set) binding to that site, we should look at the structural similarity of the designs to those antibodies, to check that we are not memorising VHHs/antibodies in the training set.

b.) We should functionally compare our designed nanobodies/scFvs to those pre-existing antibodies/VHHs, for properties such as binding affinity.

For option a), we believe we have adequately shown that we are not memorising the training set, because:

- The TcdB VHHs and scFvs bind to a site that does not have antibodies/VHHs binding to it in the PDB, and therefore cannot represent memorisation (Figures 3I-K, Figure 5).
- Extended Data Fig. 5 overlays the highest affinity designs for each site to the closest antibody/VHH in the PDB (where possible). This figure visually demonstrates high dissimilarity. Further, Fig. 2E shows sequence-dissimilarity to the training dataset, and does not demonstrate that more similar CDRs are more likely to bind.
- Given the specific request (discussed below) to look at the Influenza HA VHHs, we have included Extended Data Fig. 31 showing a zoomed-in overlay to known antibodies binding to that epitope.

For option b), we feel this is beyond the scope of this study. We were honest about the “modest affinity” of the designed VHH/scFvs, and do not claim that they would be better than the antibodies that exist in the PDB, which are generally experimentally optimized or have undergone somatic hypermutation after repeated immunizations.

3. There is significant experimental support: for example, yeast display screening of designs and then OrthoRep-enabled affinity maturation (the latter based on a method that mimics the natural evolutionary process, based on randomization and antigenic selection). As such, the reader is left wondering about the extent of innovation/improvement over current technologies. In that order, there is no indication of time investment/iteration cycles necessary to generate a possible lead VHH or ScFv. This is important because the authors claim that current methods are “laborious, time-consuming, and can fail to produce antibodies that interact with the therapeutically relevant epitope”.

We thank the reviewer for this fair criticism of the way this was presented.

We have added time estimates of the “time-to-binder” to the figure legend to Fig. 1:

“In this work, we rely on yeast surface display and/or E. coli expression + surface plasmon resonance (SPR) for experimental validation (taking approximately 6 weeks and 2 weeks post-oligonucleotide order, respectively)"

We again highlight that the innovation in this work is the **de novo** design of binding-site-specific, atomically accurate VHH/scFvs. In many cases, which we allude to in the discussion, generating antibodies/VHH to specific target proteins/binding sites is not possible with traditional methods. **De novo** design offers a potential solution in these cases.

The optimization of VHH affinity with OrthoRep is not an intellectual contribution to this work, and, as mentioned in the preamble, only aids/simplifies the downstream experimental characterisation. We do not specify a time-frame for this, as there are a plethora of alternative methods available for **antibody optimization**, some of which we do now cite in the introduction:

“There have also been recent advances in antibody optimization with deep learning methods trained on data generated by powerful new experimental methods”

This distinction is highlighted in (new) Figure 1E-F, where we highlight the full pipeline for computational antibody design (Figure 1E) and downstream experimental validation/optimization (Figure 1F).

4. The draft indicates; “In all cases, affinity-matured VHHs acquired several mutations relative to the parent designs and improved binding affinities by approximately two orders of magnitude. Thus, when the authors comment “By isolating the bound conformations, we were able to resolve the VHH-TcdB complex to a modest 5.7 Å resolution, enabling us to confidently dock the designed VHH into the cryo-EM density, confirm that the VHH folded into the expected architecture and bound to the target epitope as designed” the reader assumes that the success is also the success of affinity maturation.

We appreciate the reviewer’s concern regarding the potential confounding role of affinity maturation in interpreting the structural validation of our designed VHHs. To directly address this point, we performed additional cryo-EM analyses on the original parent TcdB VHH design (VHH_TcdB_H2), which did not undergo OrthoRep-based affinity maturation.

We reprocessed the original parent VHH/TcdB dataset and extracted a subset of particles corresponding to the parent VHH bound to TcdB. This subset was refined to 4.6 Å resolution, enabling confident docking of the parent VHH into the cryo-EM density and allowing direct structural comparison with the OrthoRep affinity-matured version from the same lineage. As expected, we observed no detectable difference in binding orientation or docking angle between the parent and affinity-matured VHHs. This result confirms that the predicted binding mode was achieved by the designed VHH alone and was not altered by the maturation process.

Note that this is also consistent with the literature. Prior work has shown that affinity maturation rarely changes the overall binding geometry, and instead primarily increases pre-organization and rigidity (see, for example, Mishra et al., 2018[1]). In that study, the conformation of the VLCDR3 loop in the CH58–V2 complex was nearly identical in the matured and unbound forms.

Additionally, not all successful structures presented in the manuscript required affinity maturation. For example, the Influenza HA VHH and the two TcdB scFvs were not affinity-matured, and their resolved structures independently confirm the accuracy of the RFdiffusion-designed binding modes without post-design optimization.

To clarify these points for the reader, we have updated the main text and now include the new cryo-EM analysis of the non-matured VHH_TcdB_H2 design. These data are presented as three additional panels in Figure 3, with updated legend text to guide interpretation.

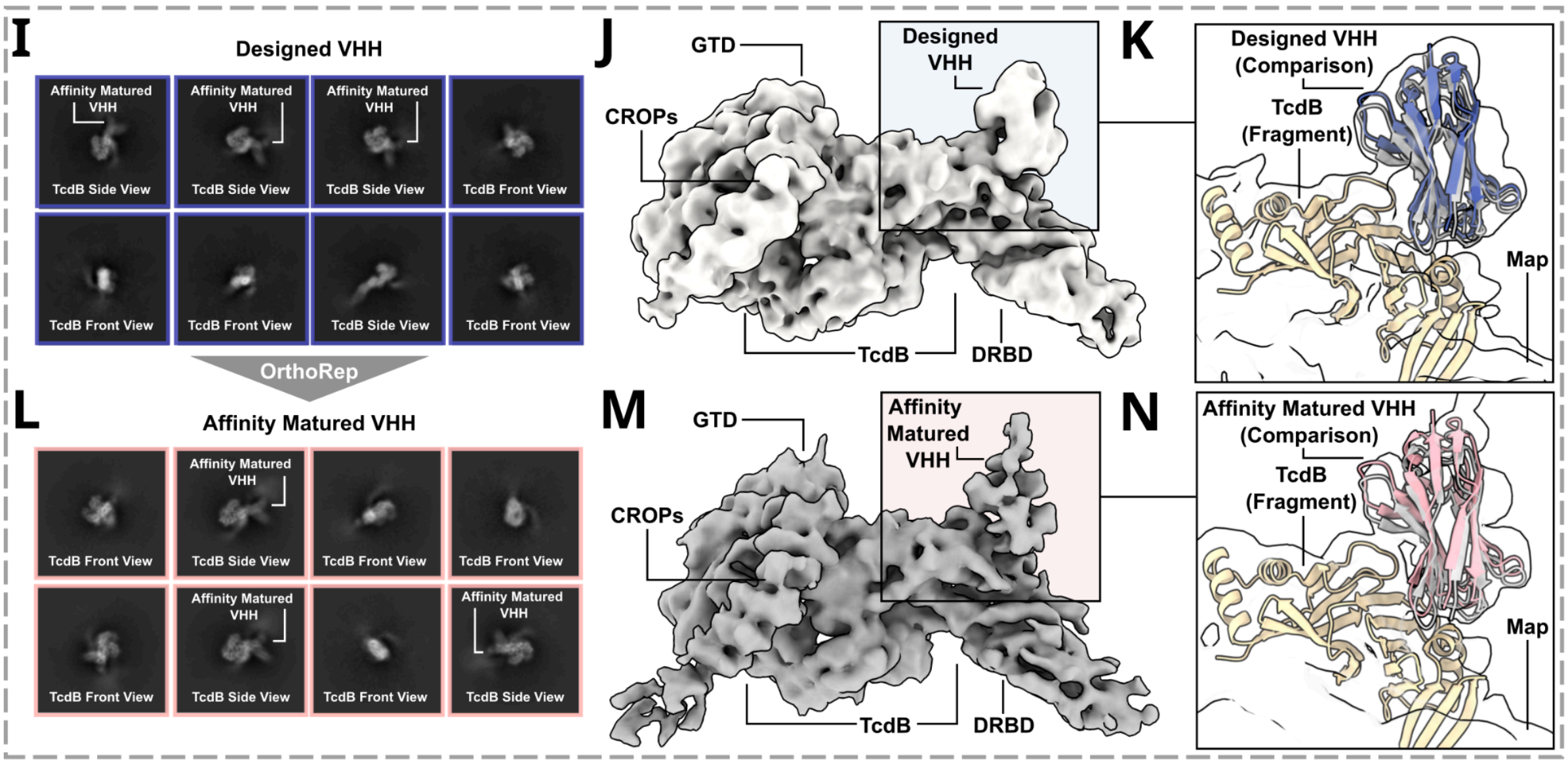

I) Labeled cryo-EM 2D class averages of the designed VHH, VHH_TcdB_H2, bound to full-length TcdB. J) A 4.6 Å cryo-EM 3D reconstruction of the complex shows VHH_TcdB_H2 bound to the target epitope as predicted. K) Due to the modest resolution, a fragment of TcdB was first docked into the cryo-EM density map, and the full design model—including both the TcdB fragment and the designed VHH—was then aligned to the pre-fitted TcdB fragment. This approach demonstrates that the predicted design closely matches the experimentally determined complex in structure, epitope targeting, and overall conformation. (Yellow = HA; navy = VHH (cryo-EM); gray = VHH (computational design prediction). M) A 5.7 Å cryo-EM 3D reconstruction of the complex shows VHH_TcdB_H2_ortho bound to the target epitope as predicted. N) As with the parent design, a fragment of TcdB was docked into the cryo-EM map, followed by alignment of the full model including the OrthoRep-matured VHH. The resulting structure shows no detectable change in binding orientation or docking angle compared to the original design, indicating that OrthoRep maturation preserved the predicted mode of epitope engagement. (Yellow = HA; pink = VHH (cryo-EM); gray = VHH (computational design prediction).

5. The work presented here differs from the original ("vanilla") RFdiffusion (and RoseTTAFold2) by fine-tuning on antibodies. Is this sufficient to claim innovation? The text also needs to spell out the contribution of other programs such as ProteinMPNN (now introduced with little detail: "After the RFdiffusion step, we use ProteinMPNN to design the CDR loop sequences") – could not find detail in the Extended Methods file either.

We strongly believe that there is sufficient innovation in this study.

Firstly, the problem itself (**de novo** design of antibodies/VHH) is a very long-standing problem in the field, and is of huge scientific and societal importance. We believe that demonstrating success on this problem for the first

time, in and of itself, is a very significant contribution. Indeed, Reviewer #3 agrees with this assessment:

"*I think it is fair to state that the authors have indeed done "atomically accurate de novo design of antibodies" as claimed by the title, in what would be the first achievement of its kind."*

"[this work considers] one of the most relevant problems in computational protein design with a clear translational application into antibody drug discovery"

"The article is technically sound and well-written, and in my opinion more than worthy of publication at Nature."

Secondly, there is significant conceptual innovation in this work. At its outset, there were not experimentally-validated, publicly-available tools for:

- Functional **de novo** antibody backbone design
- Structure-conditioned antibody sequence design
- Discrimination of successful vs unsuccessful antibody designs

In this work, we describe the necessary steps to build a pipeline for de novo antibody design, and demonstrate that this can actually design functional VHH/scFvs experimentally. We believe this describes a general framework that others can build upon to further improve de novo antibody design methods.

Thirdly, there are several technical contributions here beyond just changing the datasets:

- Conditioning on the antibody framework structure & sequence, so that we can arbitrarily choose antibody frameworks at design time. See Fig. 1B.
- The ability to sample *arbitrary* CDR lengths at design time.
- Doing this in a *global-frame-invariant manner*. This is how we can specify the VHH/scFv *framework* without specifying its rigid-body dock to the target. See Fig. 1C
- Training on additional datasets:
    - TCRs (with a distillation set of synthetic structures)
    - Loop-mediated interfaces in the PDB
- Validation of AF3 as a filter for de novo antibody design

Without these contributions, RFdiffusion (despite having antibodies in the training set), is completely unable to design antibodies/VHH (Extended Data Fig. 1).

Finally, ProteinMPNN was indeed used for sequence design in this work, unmodified from the published version. This was because ProteinMPNN was, at the time of this work, state-of-the-art for protein interface design. It is exciting to see, however, that progress beyond ProteinMPNN is possible (for example, shown very recently in Del Alamo et al., 2025[2]). Improved sequence design, paired with improved filtering (AlphaFold3), will multiplicatively improve de

novo antibody design. It is our sincere hope that the pipeline articulated and demonstrated in this work will act as the framework for further improvements, with contributions from the whole field. We believe it is an advantage of the pipeline that it is modular - backbone design, sequence design, structure prediction - which will enable swapping out individual modules for new tools as they become available.

We have added details of the usage of ProteinMPNN to the supplement, and we sincerely thank the reviewer for pointing out their omission from the original submission!

6. There is emphasis on “targeting of any specified epitope on any target of interest”. In many cases, knowledge on an epitope implies also knowledge on a paratope, ie., the pre-existence of antibodies already targeting the epitope which could be subject to improved affinity (as it is done here via OrthoRep) without starting from scratch, from zero. Importantly, throughout the manuscript there is a persistent reference to de novo design based on predefined epitopes. This terminology reflects a conceptual misunderstanding, as epitopes are inherently defined by their interaction with specific antibody paratopes. The more accurate terminology should describe a region, site, or area of the targeted antigen. Antibodies resulting from de novo design will consequently define distinct and unique epitopes within this predefined site, rather than targeting a predetermined epitope that cannot exist independently of an antibody interaction.

We agree with the reviewer that the use of the term “epitope” was somewhat imprecise in the initial submission, and agree with the formal definition of “epitope” as stated by the reviewer.

We could change the use of the word “epitope” to “binding site”. However, given that “epitope” is much more commonly used in the antibody field, we would rather leave the slightly imprecise use of the term, to aid readability.

We have, however, added clarification. We hope this will satisfy the reviewer:

Further, throughout this work, we refer to the user-specified binding site as the “epitope”, despite the epitope formally being defined by the antibody paratope.

7. Design of scFvs seems to be complex as it requires a stepwise assembly protocol to ensure the pairing between heavy and light chains. From the Extended Methods, there is also a screening step through yeast display. What is then the main contributor to success, the new computational framework, or other downstream steps in assembly for the targeting by scFvs of the user-specified epitopes identified from structure-aware designed combinatorial libraries?

Firstly, we emphasise that the stepwise assembly protocol described in Extended Data Figures 20 and 21 is indeed complicated but was necessary to achieve the scFv screening we believe is best suited to screening designed scFvs. That being said, with recent advances in gene synthesis that permit up to 500bp oligonucleotides to be synthesised (described here), the complexity required in our study, where we were limited to 300bp or 400bp oligonucleotides, can trivially be removed now using standard DNA cloning approaches.

The intellectual contribution of the assembly protocol in this work, however, is not the *specific* library assembly method, but instead how it culminates in the solution of how to test de novo designed scFvs.

There are two approaches we describe. The first (Extended Data Figure 20) describes an approach for uniquely pairing designed heavy and light chains. In contrast to random library screening approaches, where there isn't a hypothesis about which specific heavy and light chains *should* pair, random combination of heavy and light chains is standard, and is of course trivial with DNA cloning techniques. However, when one has designed a specific heavy and light chain that should pair, a strategy is needed that can assemble the *specific* heavy and light chains from an oligonucleotide pool (where the whole H-L fragment cannot be ordered in a single oligonucleotide).

We also wanted to remove the confounding non-design-related failure modes of scFvs; namely linker choice and Fv order (H-L vs L-H). The cloning strategy described in Extended Data Figure 20 achieves this, allowing multiple linkers to be tested simultaneously, and both H-L and L-H Fv orderings to be constructed from the same oligonucleotides.

The second approach, described in Extended Data Figure 21, is, in our opinion, the more significant intellectual contribution. A unique benefit of structure-based design with RFdiffusion is the *a priori* "knowledge"/hypothesis of the designed structure. Given this, it is possible to reasonably predict which specific *sets* of heavy and light chains could pair together due to their similar structures/interaction modes with the target epitope. This second approach permits the **specific pairing of *only* those heavy and light chains with compatible structure**. In other words, because we "*know"* the structure, or at a minimum have a strong structural hypothesis, we can *rationally* assemble heavy and light chains, rather than doing this randomly. Indeed, we validate computationally (Extended Data Figure 22A-B) and experimentally (Figures 4, 5) that this *specific* pairing strategy yields combinatorially-expanded libraries with high computational success rates. We also note that the highest affinity scFvs identified experimentally were from within a defined "structural cluster", but with heavy and light chains from distinct initial designs, highlighting the benefit of this design + assembly strategy. To summarise, the assembly method described in Extended Data Figure 21 permits the testing of designed scFv libraries with both the benefits of design (structural-basis/hypothesis for every scFv in the library; high success rates; every scFv designed to interact with the desired epitope)

and scale (combinatorial expansion of the library allows the testing of more scFvs).

So, to answer the question of “what is the main contributor to success”, we argue that both computational design *and* experimental screening are important, but the two are completely intertwined. We are *only* able to “intelligently” assemble scFvs with the second approach because we have a designed structure for the underlying scFv sequences enabling chain pairing between structurally compatible designs. Without design, we would be left with the traditional approach of randomly pairing heavy and light chains and *hoping* that some of those pairs yield functional antibodies that bind to a desired epitope. Note also that, while we could in theory generate *intelligently-assembled* libraries of arbitrary size, the actual sizes of the libraries tested in this work are still several orders of magnitude smaller than those typically used in random screening approaches, making the finding of multiple epitope-specific scFvs even more impressive.

8. The manuscript concludes that the method results in anatomically accurate binders. However, while attributed to the computational model, this is the result of a process where a large number of random designs in silico that are screen by HTS assays, affinity matured and then tested by SPR for binding and specificity, and then the best binder tested by cryoEM. How do the experimental steps contribute to the anatomical accuracy?

We believe we have adequately responded to this point in the preamble and earlier responses:

- The *only* contributor the atomic-accuracy is the design method (affinity maturation leaves the antibody structure essentially unchanged, as evidenced by the pre- and post-affinity maturation TcdB VHH structures now included)
- Two of the accurate high resolution structures determined in this work (H1 VHH, TcdB scFv6), as well as two of the modest-resolution structures for TcdB VHH and TcdB scFv5 did not undergo affinity maturation.

  We certainly do not claim that *every* design we make would be successful/atomically accurate. Indeed, we openly acknowledged the “quite low experimental success rates” at binding. Presumably, these unsuccessful antibodies would not be atomically accurate. The claim we make, and justify, in the manuscript is that, in most cases, when designed VHH/scFvs bind to the target, they do so in an atomically-indistinguishable manner to that which we designed with RFdiffusion. Furthermore, the size of the screens for the VHHs (96 designs for TcdB and up to 9000 for those initially screened on yeast) are orders of magnitude lower than library sizes used for isolation of VHH/antibodies from naive display libraries, implying that the design process enables far fewer designs to be tested to find on target binding precisely because they are not ‘random’.

9. The mode of binding of VHH_flu_01 to the HA stem region would merit a more detailed comparison with that of naturally isolated mAbs (such as CR6261 shown in Extended Data Fig. GH) that have shown common structural features in the interaction with the stem region, particularly with the W21 aromatic pocket (e.g., F54 and Y98 in most VH1-69 anti-stem antibodies).

We thank the reviewer for this suggestion. We have added this as Extended Data Figure 31, where we compare *VHH_flu_01* to three naturally isolated mAbs: FI6V3, CR9114, and CR6261. Here we highlight that *VHH_flu_01* does, indeed, share the structural motif of interacting with W21 through a PHE residue. This figure also highlights that *VHH_flu_01* makes this contact through a very different CDR conformation than the naturally isolated mAbs; this, in combination with the rest of the *VHH_flu_01* interface being quite distinct, demonstrates that *VHH_flu_01* is a case of *de novo* design and not simply a memorization of the training set.

10. Which is the degree of humanness of all the generated antibodies? this is a relevant aspect for their developability, as mentioned in the Discussion.

We thank the reviewer for this excellent question. In this work, we focus only on the *epitope-specific binding* part of the antibody-design problem, and do not optimize for downstream developability. This focus motivated the use of ProteinMPNN, rather than an antibody-specific sequence design method. As such, the humanness of designed VHHs is somewhat lower, as assessed by OASis Identity[3], than therapeutic antibodies (pink vs blue):

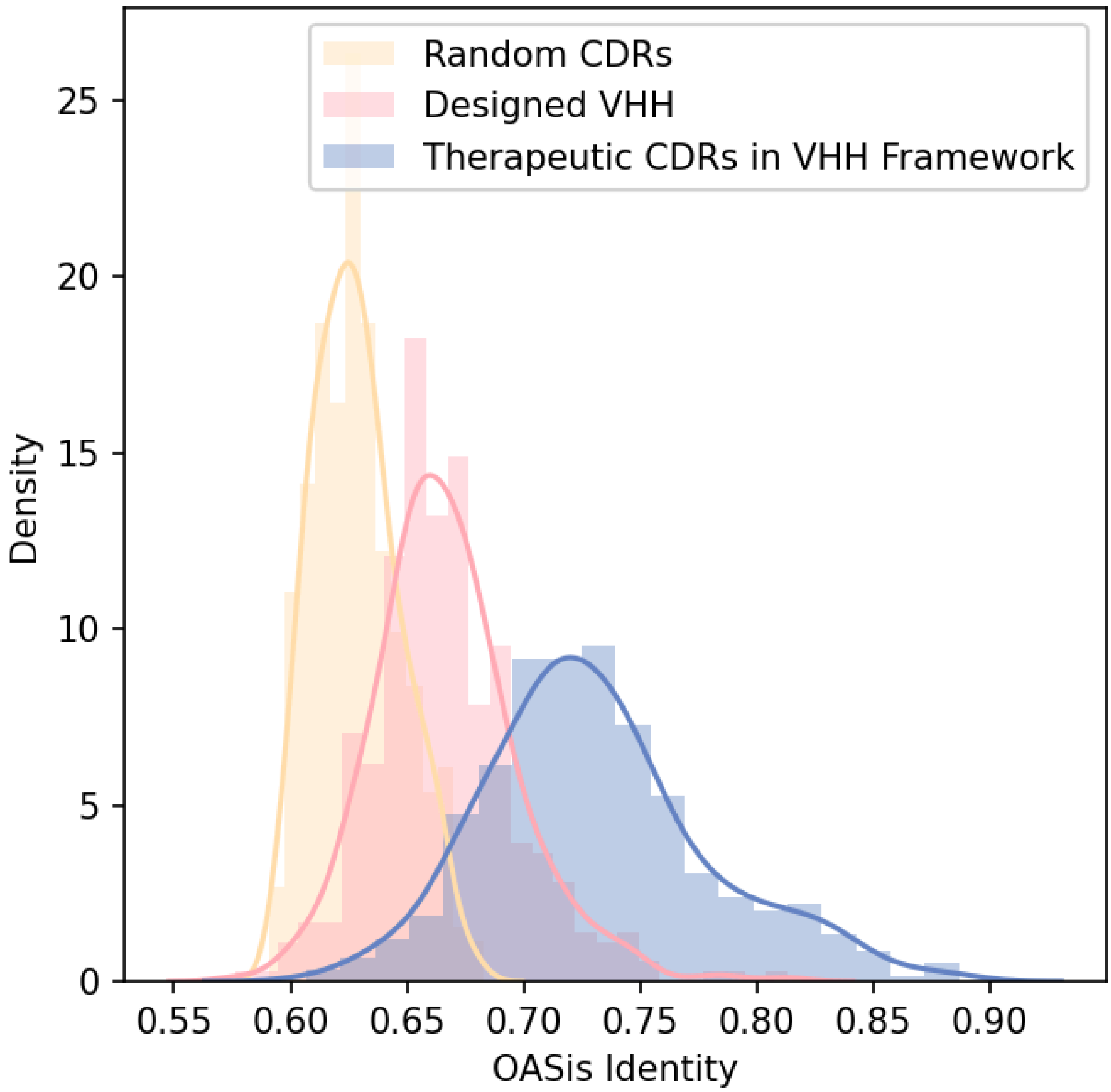

We attribute this reduced humanness to the use of ProteinMPNN, which trains on the full PDB. As a control, we sampled “random” CDR sequences using the distributions of amino acid frequencies in the human proteome, to approximate the distribution that ProteinMPNN has trained on. Encouragingly, designed VHH are more human than these random sequences (pink vs yellow), implying that the RFdiffusion-designed CDR *structures* are CDR-like enough to imply a more CDR-like/human sequence.

We leave the optimization of humanness, and other developability properties, to future work.

This figure has been added to Extended Data Figure 1, and the main text makes reference to this:

“Indeed, designed sequences are currently somewhat less human (as assessed by OASis score) than therapeutic antibody CDRs (Extended Data Fig. 1D).”

11. Other points:
Discussion: would be good to comment on other approaches to solve the design of antibodies: example https://pubmed.ncbi.nlm.nih.gov/39793083/, https://www.biorxiv.org/content/10.1101/2024.11.11.622872v1, https://arxiv.org/abs/2306.12360 https://arxiv.org/abs/2409.08022

While we have included extensive citations to other work, we believe the references suggested by the reviewer are inappropriate to add. We justify this assessment in the order provided by the reviewer:

1. Singh, R. *et al*., 2025. This describes an approach for **antibody optimization**. They demonstrate this approach elegantly for **optimizing** pre-existing antibodies to SARS-CoV-2. In other words, this is not **de novo** antibody design.
2. Agarwal, A. A. *et al*., 2024. This extremely powerful approach again tackles the problem of **antibody optimization**, rather than de novo design. Indeed, AlphaBind generates optimized variants no more than 11 mutations for a pre-existing binding antibody. However, given how powerful AlphaBind is for antibody optimization, we do now cite this in the introduction as one of the possible ways of performing downstream affinity maturation:
    a. *"There have also been recent advances in antibody optimization with deep learning methods trained on data generated by powerful new experimental methods"*
3. Frey, N. C. *et al*., 2023. This again tackles **antibody optimization**, rather than de novo design.
4. Zambaldi, V. *et al.,* 2024. AlphaProteo generates **de novo** interfaces, but these are miniproteins. They do not design antibodies/VHH in this study. There are also no model details or available code.

We do however now make reference to JAM, from Nabla Bio, who, subsequent to this work, announced experimental success at VHH design (although do not release any model details or code):

"Ultimately, computational de novo design of antibodies using our RFdiffusion and related approaches (such as the recently announced JAM model) could revolutionize antibody discovery and development."

Figures: Fig 1 – there is an implicit assumption that RFdiffusion needs a target structure and epitope for design. Please discuss.

There are two aspects to this point. We answer these in turn.

"RFdiffusion needs a target structure". This is indeed the case, and is also the case for most published binder design methods[4–6]. An alternative approach would be to *predict* the target structure while generating a binder to it. This has been done with some success both by us for peptide binder design[7] and by

others for de novo miniprotein design[8]. However, given that interface design requires very high resolution and accuracy interaction design, and that modeling of loop-mediated interfaces is particularly challenging, in this work, we chose to design VHH/antibodies against known high-resolution structures. This is likely particularly important for the epitope on TcdB, which is inherently very flexible and therefore challenging to model accurately. While it will be interesting to further this technology towards the design of VHH/antibodies against *predicted* (i.e. unsolved) structures, this is beyond the scope of the current work.

"[RFdiffusion needs an] epitope for design". The key benefit of de novo VHH/antibody design is the ability to specify the epitope to which an antibody interacts to a target. We therefore see the specification of the epitope as a "luxury" rather than just a "necessity". During training, we mask 80-100% of interacting residues on the target protein. Therefore, some fraction of the time, the network *does not* receive epitope information. It should therefore be possible to *not* provide epitope information at inference time, but, given that this is *not* how a user would typically want to use RFdiffusion, we leave this analysis for future work.

Minor

1. As indicated above, the manuscript would benefit from a schematic figure presenting the steps of the VHH or scFvs design (computing and experimental steps)

We agree with the reviewer that this is a very helpful addition to the paper, and we have now added this in Fig. 1E-F. So as to be as explicit and helpful to the reader as possible, we have also acknowledged and suggested the use of AlphaFold3 as the "filtering" model in the figure legend.

2. "Instead, antibody discovery currently relies on animal immunization or random library screening approaches." - this sentence omits the frequent identification of antibodies from human memory B cells or plasma cells which are recognized as the optimal source for the isolation of anti-infective antibodies.

We thank the reviewer for this suggestion, which we have incorporated into the text:

"Instead, antibody discovery currently relies on animal immunization, random library screening approaches, or the isolation of antibodies directly from patient B cells."

3. The directed evolution platform OrthoRep is not defined nor referenced in the abstract (https://www.nature.com/articles/s41467-020-19539-6)

As aforementioned in the preamble, the key contribution of this work is the epitope-specific design of binding antibodies, not in the affinity maturation or

downstream optimization of initial designs. In this study, purely to permit the solving of high resolution structures (the basis of our "atomically accurate" claim), we performed affinity maturation of our initial designs. We used OrthoRep, but we could, in theory, have used any existing method for this step.

Therefore, as it is not part of the contributions of this work, we would rather omit it from the abstract, as it does not constitute a key result/contribution. However, for the reader who may be unfamiliar with the OrthoRep system, we have added a little more explanation as to the method, rewording:

"we utilized the OrthoRep system for continuous hypermutation of target genes in vivo"

to:

"we utilized the orthogonal error-prone DNA replication system, OrthoRep, for continuous hypermutation of target genes in vivo."

4. The following comment in Introduction seems counterintuitive, as it seems to indicate that it is positive that there are few antibody sequences in PDB : "RoseTTAFold2 (RF2) and RFdiffusion are trained on the entire Protein Data Bank (PDB16) which helps overcome the problem that the PDB contains relatively few antibody structures (~8,100 antibody structures versus >200,000 total structures), complicating the training of large machine learning models.

We thank the reviewer for pointing out this somewhat confusing wording. The intent was to highlight that it is a "problem" that there are "relatively few antibody structures", but we have rephrased this for clarity.

"RoseTTAFold2 (RF2) and RFdiffusion are trained on the entire Protein Data Bank (PDB, > 200,000 total structures) which helps overcome the problematic limited availability of antibody structures (~8,100)"

5. The authors hint at how AlphaFold3 would fit in the current protocol. In particular because of the comment that "A key contributor to previous successes in de novo binder design was improved filters (primarily AlphaFold244), which enriched for experimental success in the subset of designs that are tested experimentally9,20. At the outset of this study, we sought to build such a filter by fine-tuning RoseTTAFold2 (Extended Data Figs. 2-4), but the filtering power of this model is limited". It would be useful to have a comment regarding the results of Hitawala and Gray (ref 45).

While we have included reference to Hitawala and Gray, we agree there is nuance that was not provided in our original phrasing. We have extended the reference to:

"Subsequent to the design work in this study, AlphaFold3 was released that has improved antibody structure prediction accuracy [22,45], both with [22,45] and without [45] antigen present."

6. The manuscript indicated that "The CDR loops are distinct from VHHs observed in nature, indicating significant generalization beyond the training dataset" – an alternative statement is that CDR diversity is just reflecting hallucination. The statement would be stronger if referring to successful binders.

We thank the reviewer for pointing out the omission of any comment on the similarity of "successful" binders. We had highlighted that successful binders are no more similar to the training dataset than unsuccessful binders in Fig. 2E (red lines), but we now also reference this in the text:

"Furthermore, no correlation between training dataset similarity and binding success was seen (Fig. 2E, red lines)."

7. What is the size of libraries in steps such as: "We succeeded in identifying epitope specific scFvs from the heavy-light combinatorial libraries".

We agree this is helpful to highlight. We have added a reference in the main text to this value and the figure showing it:

"We succeeded in identifying epitope specific scFvs from the heavy-light combinatorial libraries (of a theoretical complexity of approximately 10M, Extended Data Fig. 22B-C) but not the fixed pairing libraries (Extended Data Fig. 23)."

8. The authors make a passing comment in Discussion that "A structure-based approach to antibody design enables the optimization of key pharmaceutical properties, such as aggregation, solubility, and expression levels, in a structurally informed manner." This risks to be interpreted as an easy and logical task – but it is far from simple.

We agree this sentence could certainly have been misinterpreted, which was not our intention. We have toned this down, to:

"A structure-based approach to antibody design may also aid the optimization of key pharmaceutical properties, such as aggregation, solubility, and expression levels (all major challenges in antibody development), in a structurally informed manner."

9. It is not very clear from the text which is the "intended epitope" against which the RBD VHH were generated against.

In Extended Data Figure 17, we show the intended dock of the designed VHH overlaid with the experimental structure, which highlights the intended epitope/dock orientation vs that which was actually achieved. For clarity, we

have added language to the corresponding figure legend specifying the specific residues targeted during the design process (which were previously provided only in the supplement).

"... VHH designed to bind hotspot residues 492-497 (PDB: 6M0J)"

We also highlight Extended Data Fig. 17F-H, where the overlay of the design model with the subsequent Cryo-EM structure is shown:

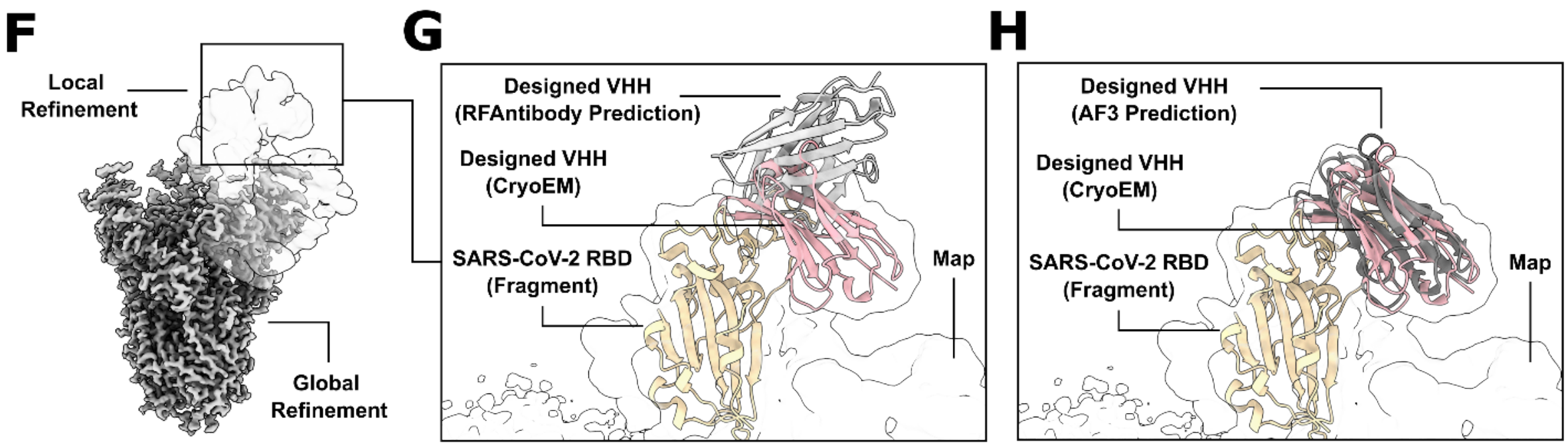

10. In Extended Data Figure 5 E and H the provided PBD code (6YFT) is wrong (it is referred to the VLP of Wenzhou levi-like virus 1).

A huge thank you for an excellent catch! This has been corrected to **6FYT**

11. In Extended Data Figure 8, the order of the panels should be grouped by targeted antigen. The current order s

We thank the reviewer for pointing this out. We have reorganised this figure.

Referee #3 (Remarks to the Author):

The paper by Bennett, Watson et al. addresses one of the most relevant questions in modern computational protein design: the development of methods to design antibodies in silico that exhibit high potency and bind to specific epitopes. The authors develop a fine-tuned version of their early tool, RFDiffusion, to generate de novo antibodies with a diffusion protocol; and a fine-tuned version of their also earlier tool, RoseTTAFold 2, which is used to select promising designs with high success. The authors use these tools to design single-domain antibodies against various proteins, and later also scFvs. The antibodies designed by the authors exhibit potencies in the double- and triple-digit nanomolar range, which can generally be optimized by standard affinity maturation to a level of affinity that enables structural characterization by CryoEM. Indeed, the experimental structures show, in most cases, an uncanny resemblance to the designs. The exposition of results is quite convincing, and I think it is fair to state that the authors have indeed done "atomically accurate de

novo design of antibodies" as claimed by the title, in what would be the first achievement of its kind.

We sincerely thank the reviewer for this summary and assessment of the work!

This work is one of the most interesting articles I have ever read, considering one of the most relevant problems in computational protein design with a clear translational application into antibody drug discovery. The contribution to the literature is obvious and very significant, and I expect broad interest from those working in antibody drug discovery, but also in protein science, in general. The article is technically sound and well-written, and in my opinion more than worthy of publication at Nature. I am delighted to recommend that the article is published.

We again thank the reviewer for recommending the work for publication, and for highlighting both the importance of the problem and for complimenting the technical quality of the work!

I would however like to request that the authors address a few questions before the article is fully accepted for publication:

- In the abstract, the authors state that initial computational designs exhibit "modest" affinity — could I suggest that they specify the actual affinity levels i.e. around triple-digit nanomolar?

We agree this is helpful for the reader, and as such have added the suggestion:

"While initial computational designs typically exhibit modest affinity (tens to hundreds of nanomolar K_D),"

- The final section constitutes, in my opinion, one of the most thought-provoking parts of the paper, in particular the authors' claim that, had AlphaFold 3 been available, the success rate of experimental designs would have been notably increased. I think this raises the interesting question of exactly how good the fine-tuned version of RoseTTAFold 2 is at predicting and filtering antibody structures. Would the authors be able to compare Extended Figures 3 and 4 to un-tuned RF2, and naïve AF2 and AF3?

Thank you for highlighting the interest in this section, which we provided to both provide perspective as to where this design pipeline will go in the future, and to help users to use better filters when they use RFdiffusion to design de novo VHH/scFvs.

The precise question is somewhat tricky to answer. AF2 and RoseTTAFold2 are *very* bad at predicting antibody complex structures, and this is generally due to challenges in finding the global dock, without co-evolutionary information. Because the rates of predicting the correct complex structure in these models (in contrast to AlphaFold3) are so low, we cannot hope to use

their “confidence” metrics as a reliable predictor of experimental binding. We sought to overcome this by making the problem simpler: we fine-tuned RF2 providing both the target structure and (some of) the epitopes to which the antibody was designed/known to bind (see below for a more in-depth discussion). This means that a lot of antibody structures can be accurately predicted (e.g. with 100% of epitope residues provided, around 50% of antibody structures can be accurately and confidently predicted; Extended Data Fig. 2B). However, this is obviously *mostly* due to the additional provided information.

AlphaFold3 often does not require such privileged information to accurately predict protein structures, so it *is* possible to use it as a design filter. However, it is hard to do a head-to-head comparison with the fine-tuned RoseTTAFold2 model, because of the fact that essentially all of the designs we have data on have already been filtered with the fine-tuned RoseTTAFold2. Therefore, by definition, they have high-confidence RoseTTAFold2 scores.

However, the very impressive ROC curves we derived with AlphaFold3 (Extended Data Fig. 18), even despite the fact that designs had been pre-filtered with the fine-tuned RoseTTAFold2 network, indicates that it is a significantly better design filter, which we try to make extremely clear to the reader/user.

To provide some data to compare the models suggested by the reviewer, however, we extend the analysis of antibody *monomer* prediction to include all of these models (Extended Data Fig. 3B), where we see that, as expected, the fine-tuned RoseTTAFold2 network is slightly better than AF2 (and IgFold), but slightly worse than AF3 (see below).

- The comparison in Extended Figure 3 is only done against IgFold, which is one of the available methods and not always the most accurate. Would the authors be able to also compare against IgFold? I would also appreciate if the figures could report average RMSDs of both methods, rather than just Wilcoxon tests.

The precise ask here is unfortunately unclear because of a presumed typo, but we have now added comparisons to AlphaFold2 and AlphaFold3 to Extended Data Fig. 3 (and reproduced below), which is likely the most relevant comparison.

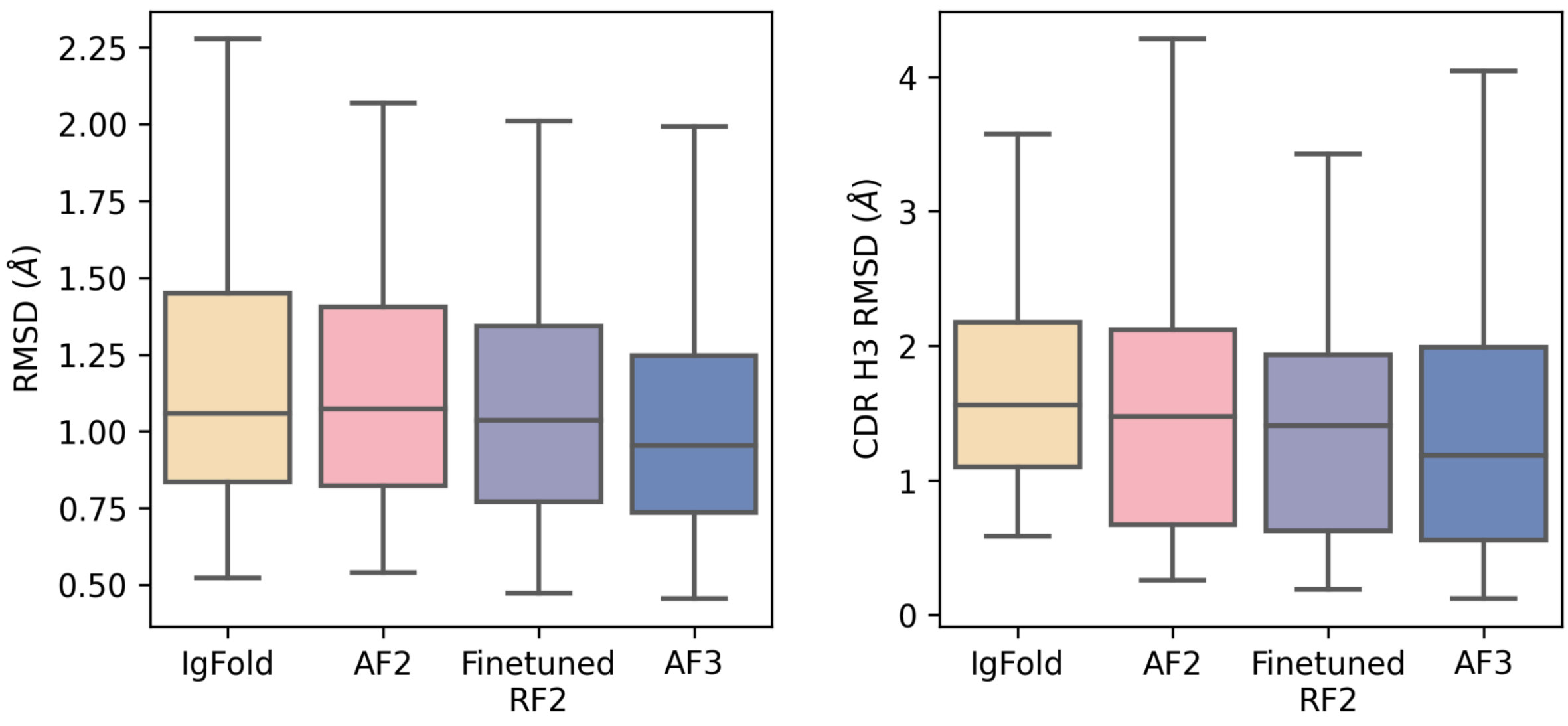

- Why does fine-tuned RF2 work so well on antibody structures? The prevailing view about antibody structure prediction has been that, since deep learning models rely on processing the MSA for evolutionary contact information and implicitly projecting it into 3D space (see e.g. the OpenFold paper by M. AlQuraishi, or the papers by S. Ovchinnikov), and antibodies do not have an evolutionary story, predicting CDR structures is hard. The authors seem to solve the problem by fine-tuning on a specific database (SAbDab + TCR structures + mined loop-target structures from the PDB), and a set of negative examples (mismatching targets + swapping CDRs + an auxiliary yeast display test set). Intuitively, I would have argued that the first approach would have little impact because the structures should already be in the training set of RF2/AF2/AF3, and that mismatching targets or swapping CDRs would have a limited example. Would the authors be able to provide some ablation study (even on just a couple of epochs, to see tendencies) of how much each of these approaches contribute to the significant improvement in RF2?

This is an excellent question, and we break it into two parts; structure prediction of *unbound* and *bound* antibodies.

As shown in the original submission, fine-tuning RoseTTAFold on antibody structures leads to slightly better performance at unbound antibody structure prediction than IgFold. We attribute this to RoseTTAFold likely being a slightly more performative architecture, and on RoseTTAFold training on more structural data (the full PDB, an AlphaFold distillation set), as well as fine-tuning on a broad range of loop-mediated datasets (TCRs, loop-mediated interfaces, antibodies). We did not want to overstate this improvement, however, as it is not dramatic, and, as we now show, AlphaFold3 outperforms fine-tuned RoseTTAFold on this task.

For antibody complex prediction, there are two key answers that were stated in the text, but we recognise were not as clear as it could have been. We stated:

“With pAE < 10, 80.3% of structures are within 2 Å when 100% of “hotspots” are provided (along with the holo target structure), with this falling to 52.6% when only 10% of hotspots are provided.”

“Accuracy is higher when the bound (holo) conformation of the target structure is provided”

In other words, 1) fine-tuned RoseTTAFold is only very accurate when (some of) the interface is provided as additional information to the network, and 2) the “holo” structure (with the induced-fit imprinted in it), makes the problem of antibody complex structure prediction significantly simpler. During design, we know the epitope to which the antibody was designed to bind, and we have a (typically) crystal structure that we are designing against, so, in effect, we always have the epitope and “holo” structure during structure prediction-based filtering. This was the rationale for fine-tuning RoseTTAFold in the manner that we did; to make it useful for design filtering.

For traditional (i.e. non-design related) antibody structure prediction, hotspot information is not available unless determined experimentally. Therefore, we made no claims about the ability of fine-tuned RoseTTAFold to do *actual* structure prediction and is instead used as a design tool.

There is significant active work to try to improve conventional structure prediction models (such as AlphaFold3) on antibody structure prediction, through augmenting training datasets and improving training regimens, and as such, we leave more detailed analysis to future work. We have, however, tried to be more explicit about what the fine-tuned RoseTTAFold model is (a filtering model, *not* a structure prediction model):

“We sought to build an improved design filter by fine-tuning the RoseTTAFold2 structure prediction network on antibody structures. To make the problem more tractable, we provide information during training about the structure of the target and the location of the target epitope to which the antibody binds; the fine-tuned RF2 must still correctly model the CDRs and find the correct orientation of the antibody against the targeted region. The rationale for providing this additional information is that the target structure and binding location are available during design (but are typically not during general structure prediction). With this training regimen and additional information, RF2 is able to robustly distinguish true antibody-antigen pairs from decoy pairs and often accurately predicts antibody-antigen complex structures. This is notably only the case when the bound (holo) conformation of the target structure and epitope information is provided (Extended Data Fig. 2).”

- On that note, could the authors provide more information about the yeast display data they use in model fine-tuning (section 5.12 of the extended data)?

How much data was generated, and how relevant is this for the final performance of the model?

This is an excellent question. We have added this to supplementary section 5.12:

“The yeast display data was generated from many minibinder design campaigns performed using the protocol described in Cao et al.[18], Bennett et al.[24], and Watson et al.[1]. The yeast surface display experiments were performed in the manner described in Cao et al.[18] and the designs are classified as binder or non-binder according to the criteria proposed in that work. The final dataset used for training consisted of 1.6M designs against 41 different target proteins.”

Several of the sets which comprise the larger dataset have been publicly released, including the sets from Cao et al.[4], Bennett et al.[9], and Watson et al.[5]. The remaining sets are the work of many different people and are not released with this publication.

- Finally, I would ask if the authors could report an estimate of the GPU resources required to finetune RF2 and RFdiffusion, so as to estimate the amount of FLOPs required for future research.

We thank the reviewer for this important question. We have added this to supplementary section 3.6 (newly added text in **bold**):

“*RFdiffusion was trained for 70 epochs with 512 examples per epoch**, this took ~3 days on 2 NVIDIA 80GB A100s (80GB of GPU memory is required to train on the larger crop size of 768)**.*“

And supplementary section 5.15:

“*RF2 was trained for 400 epochs with 512 examples per epoch**, this took ~7 days on 8 NVIDIA L40s.** All other training settings used are the same as in the published RF2.*”

“For this stage, we trained for 207 epochs with 512 examples per epoch**, this took ~3 days on 4 NVIDIA L40s**.”

On a side comment, the code provided by the authors in the submission appears essentially identical to the public GitHub repository for RFdiffusion 1.1, rather than the RFantibody repository -- was this intended? I would ask if the authors are planning to release any code on the training code used to fine-tune the base models.

We sincerely apologise for providing the *wrong* link to the GitHub repository. This is now fixed:

https://github.com/RosettaCommons/RFantibody

We note that this code is fully commercially available.

In line with previous papers[5,10,11], both from our lab and others, we do not plan on releasing training code, as it is sensitive for a number of ongoing projects, and is the work of people beyond this project.

We have, however, shared the antibody training dataset on Zenodo (https://zenodo.org/records/15741710), to permit reproducibility of, for example, training and validation splits.

I would like to conclude by commending the authors for the extraordinary achievements reported here, and for their commitment to open science by disseminating them in this article.

Thank you sincerely Carlos, for the highly insightful and helpful review of the paper!

Carlos Outeiral

Referee #3 (Remarks on code availability):

The code provided by the authors appears essentially identical to the public GitHub repository for RFdiffusion 1.1, rather than the RFantibody repository.

We sincerely apologise for providing the *wrong* link to the GitHub repository. This is now fixed:

https://github.com/RosettaCommons/RFantibody

Referee #4 (Remarks to the Author):

In this study, the authors report de novo antibody design using a fine-tuned RFdiffusion model combined with experimental screening and optimization. The authors demonstrated the ability to generate functional VHHs and scFvs targeting disease-relevant epitopes. Several of the designs were confirmed with cryo-EM structures. The biological relevance of the case studies is compelling, particularly the targeting of the conserved HA stem epitope and the Frizzled-7 interface of TcdB, which lacks existing antibody structures. The study clearly represents a significant advance in computational antibody design.

However, several issues need to be addressed to better reflect the pipeline's strengths and limitations, enhance its reproducibility, and accelerate adoption by relevant communities.

1.The current manuscript lacks a figure that visually summarizes the overall computational and experimental pipeline, requiring readers to piece together the steps from the text through which the authors achieved their final goals. The current Figure 1 focuses only on the computational aspects and does not adequately represent the broader workflow. Furthermore, panels B to D of Figure 1 are highly repetitive, and the straightforward progression between them has already been clearly presented in the main text. It can be very helpful to replace these panels with a multi-panel schematic figure to outline the key steps, from epitope-targeted CDR design to experimental screening and affinity maturation.

We thank the reviewer for this suggestion. We have added Figure 1E-F, where we highlight the full pipeline for computational antibody design (Figure 1E) and downstream experimental validation/optimization (Figure 1F). We agree with the reviewer that this clarifies the pipeline used in this work. We believe Figure 1B-D highlights some of the key methodological contributions of this work and we have therefore kept these panels, as it is possible to add the detailed pipeline figure without having to remove them.

2. Additional tables or plots are needed to highlight important results regarding the intrinsic accuracy and success rate of the design protocols, as well as the efficiency of the computational and experimental filtering strategies. Example data to include in these tables might consist of: the number of initial RFdiffusion designs per target, the fraction retained after RF2 filtering (with pAE/iPTM thresholds), hit rates in experimental screening, affinity maturation success rates (e.g., fold-improvement), etc..

We have added Supplementary Methods Table 6 which summarizes the experimental and computational success rates at each step for the VHH design campaigns which should better describe to the reader the computational and experimental success rates for each step in the design pipeline.

3. The fine-tuning of RFdiffusion and RoseTTAFold2 is central to this work. However, the currently released repository lacks the training/fine-tuning code and datasets. Releasing these resources, especially the datasets, can enhance the reproducibility of the study and facilitate benchmarking against alternative methods.

We agree with the reviewer that a key contribution of this work is the open-source release of the models we developed. In keeping with standard practice in the field[5,10,11], we have released only the inference code for these

models. Additionally, we have made sure there are no restrictions to their use (either by academics or industry), to maximise their benefit. We do not plan to release training code, as it is sensitive for a number of ongoing projects, and is the work of people beyond this project. We do, however, very extensively describe the training protocol in the Supplement (sections 3 and 5).

Additionally, we have the antibody training dataset on Zenodo (https://zenodo.org/records/15741710), to permit reproducibility of, for example, training and validation splits.

References

1. Mishra, A. K. & Mariuzza, R. A. Insights into the Structural Basis of Antibody Affinity Maturation from Next-Generation Sequencing. *Front. Immunol.* **9**, 117 (2018).
2. Adapting ProteinMPNN for antibody design without retraining.
3. Prihoda, D. *et al.* BioPhi: A platform for antibody design, humanization, and humanness evaluation based on natural antibody repertoires and deep learning. *mAbs* **14**, 2020203 (2022).
4. Cao, L. *et al.* Design of protein-binding proteins from the target structure alone. *Nature* **605**, 551–560 (2022).
5. Watson, J. L. *et al.* De novo design of protein structure and function with RFdiffusion. *Nature* **620**, 1089–1100 (2023).
6. Gainza, P. *et al.* De novo design of protein interactions with learned surface fingerprints. *Nature* **617**, 176–184 (2023).
7. Vázquez Torres, S. *et al.* De novo design of high-affinity binders of bioactive helical peptides. *Nature* **626**, 435–442 (2024).
8. Pacesa, M. *et al.* BindCraft: one-shot design of functional protein binders. Preprint at https://doi.org/10.1101/2024.09.30.615802 (2024).
9. Bennett, N. R. *et al.* Improving de novo protein binder design with deep learning. *Nat. Commun.* **14**, 2625 (2023).

10. Jumper, J. *et al.* Highly accurate protein structure prediction with AlphaFold. *Nature* **596**, 583–589 (2021).
11. Abramson, J. *et al.* Accurate structure prediction of biomolecular interactions with AlphaFold 3. *Nature* **630**, 493–500 (2024).
